# Comparison of drug safety data obtained from the monitoring system, literature, and social media: An empirical proof from a Chinese patent medicine

**DOI:** 10.1371/journal.pone.0222077

**Published:** 2019-11-06

**Authors:** Ruixue Hu, Su Golder, Guoyan Yang, Xun Li, Di Wang, Liqiong Wang, Ruyu Xia, Nanqi Zhao, Sainan Fang, Baoyong Lai, Jianping Liu, Yutong Fei

**Affiliations:** 1 Centre for Evidence-Based Chinese Medicine, Beijing University of Chinese Medicine, Beijing, China; 2 Department of Health Sciences, University of York, York, England, United Kingdom; 3 NICM Health Research Institute, Western Sydney University, Penrith, NSW, Australia; 4 School of acupuncture-Moxibustion and Tuina, Beijing University of Chinese Medicine, Beijing, China; Auburn University, UNITED STATES

## Abstract

**Objectives:**

To investigate the consistency of adverse events (AEs) and adverse drug reactions (ADRs) reported in the literature, monitoring and social media data.

**Methods:**

Using one Chinese patent medicine-Cordyceps sinensis extracts (CSE) as an example, we obtained safety data from the national monitoring system (July 2002 to February 2016), literature (up to November 2016) and social media (May 2019). For literature data, we searched the Chinese National Knowledge Infrastructure Database (CNKI), WanFang database, Chinese Science and Technology Periodical Database (VIP), Chinese Biomedical Literature Database (SinoMed), PubMed, Embase and the Cochrane Library. Social media data was from the Baidu post bar and Sina micro-blog. Two authors independently screened the literature and extracted data by PRISMA Harms checklist was followed. AEs and ADRs were coded using the World Health Organization Adverse Reaction Terminology (WHO-ART). AEs and ADRs were grouped into thirty-one organ-system classes for comparisons. Frequencies, relative frequencies and rank were used as metrics. Radar chart was used to manifest the features of the distributions and proportions.

**Results:**

610 AEs reported in CFDA monitoring data were associated with CSE, of which 537 (88.03%) were suspected ADRs (10.49% certain). 5568 AEs were identified from 172 papers (63% RCTs, 37% other types of studies including case series, case reports, ADR monitoring reports and reviews), in which 86 (1.54%) were ADRs (1.54% certain). 15 AEs (0 certain ADR) were identified from social media. AEs, ADRs and their affected system-organ classes, looked largely similar, but different in every aspect when looking at details. Data from RCTs demonstrated the most disparity.

**Conclusions:**

In our study, the most prevalent AEs and ADRs, mainly gastro-intestinal system disorders including nausea, diarrhea and vomiting, in monitoring system were largely similar with those in literature and social media. But data from different sources varied if looked at details. Multiple data sources (the monitoring system, literature and social media) should be integrated to collect safety information of interventions. The distributions of AEs and ADRs from RCTs were least similar with the data from other sources. Our empirical proof is consistent with other similar studies.

## Introduction

Guideline developers, clinicians and patients need to balance the safety and effectiveness of interventions when making clinical recommendations or decisions. Adverse events (AEs) and Adverse Drug Reactions (ADRs) are measures to observe and study safety. An AE is a negative medical event which occurs when using a medicinal product, but not necessarily causally related [1]. ADR is a response to a drug that is noxious and unintended and occurs at doses normally used in people for the prophylaxis, diagnosis or treatment of diseases, or for modification of physiological functions [2].

The clinical safety of interventions can be observed through the national or international monitoring systems. The Uppsala Monitoring Center (UMC) was the first World Health Organization (WHO) Collaborating Centre established for international drug monitoring in 1978, which is an agency under the WHO, responsible for collecting reports on ADRs [3], summarizing and categorizing the reported cases, and providing feedback to health care providers [4]. Voluntary reporting system for ADRs is a basic method adopted by most members of the WHO International Drug Surveillance Program, which has a wide range of monitoring systems, is less costly with good reputation, and makes it more possible to capture rare or new AEs and ADRs [5]. China is also a member of UMC. The Chinese Food and Drug Administration (CFDA), now changed to the National Medical Products Administration (NMPA) of China, established an ADR online reporting system in 2004 and all the hospitals and clinics are required to report AEs and ADRs via this system [4].

Scientific literatures and social media are also important information source for drug safety reporting. People may choose to publish papers to report safety findings or to speak up in the open social media about their AEs/ADRs, rather than reporting them to the monitoring system [6]. All types of clinical research literature can provide important information for AEs/ADRs of interventions. Safety observation is compulsory in randomized controlled trials (RCTs) [7]. Observational studies with large sample sizes and long-term follow-ups [8–11], such as cohorts and registry studies, are considered as validated study designs for the evaluation of safety of interventions. There is also important information of AEs/ADRs in case reports. Previous study showed that case reports accounted for 71.4% of the reported ADRs, which caused post-marketing withdrawal of medicinal products [12]. In China, Baidu post bar and Sina micro-blog like twitter are the most popular open public social media tools where people can express their own attitudes to the public [13–15]. AEs or ADRs are also likely to show up in such social media tools.

However, the data collected by the national monitoring systems is often unavailable to most researchers and the public. Also, real-world studies like large scale observational studies and post-market surveillance studies are often lacking for many interventions [16], especially in the traditional Chinese medicine field [17–19].

Facing such challenges, we need to find a better solution for researchers to adequately evaluate the safety of interventions, especially when lacking large scale observational studies and national monitoring data. We aim to explore whether the evaluation of safety based on published and unpublished literature and social media is consistent with the data of monitoring systems or not, and is it necessary to integrate all data source for the evaluation of drug safety.

In 2016, we were commissioned by the CFDA to systematically review the safety of one Chinese patent medicine-Cordyceps Sinensis Extracts (CSE), using the safety data from CFDA monitoring system and the published and unpublished literature. The main pharmacological action of CSE is similar to natural Cordyceps Sinensis which has anti-inflammatory, antitussive, expectorant, sedative and gonadotropic effects mainly for chronic bronchitis, chronic renal insufficiency and hyperlipidemia [20]. The CSE was included in the National Health Insurance Catalogue [21] and the Chinese Pharmacopoeia [22]. It was also recommended in two clinical practice guidelines [23,24]. And it belongs to the top 100 medical products with science and technology of big brand traditional Chinese medicine. [25]. This project provided an opportunity for us to explore this methodological issue with sufficient data.

## Material and methods

Monitoring data of CSE was searched by CFDA professionals and given to us in its clean form (File A in S1 Appendix). The data included 57 items: anonymous patient demographic information (8 items), patient historical ADR information (3 items), patient health condition (1 item), and description of AEs (11 items), medications received (19 items), causal inference judgments of AEs (6 items), and reporter information (9 items). We obtained the AEs and ADRs data of CSE reported during July 2003 to February 2016. There was no duplicated record in the obtained the monitoring data.

We searched the published literature from major Chinese and English databases: the Chinese National Knowledge Infrastructure Database (CNKI), Wanfang database, Chinese Science and Technology Periodical Database (VIP), Chinese Biomedical Literature Database (SinoMed), PubMed, Embase, and the Cochrane Library (Central database and Database of Abstracts of Effects, DARE) from their inception to November 2016. Unpublished literature (including conference proceedings and academic degree dissertations unpublished were also achieved via searching through the four Chinese databases) (File B in S1 Appendix). Literature obtained included all types of empirical clinical studies that used CSE to treat diseases, alone or in combination with any other medication. We excluded animal studies, and studies that did not mention any word or phrase relevant to safety. Followed with PRISMA Harms checklist [26–29], two authors independently screened (title and abstract screening and full text screening) and extracted data. Any disagreement regarding study selection and data extraction was resolved through discussion if necessary. Literature was classified by study type to be RCTs and other types of studies.

We also searched social media data (the articles, topics and posts) in Baidu post bar related to CSE [30] and the Sina micro-blog websites [31]. All the contents of the post bar and the blogs were saved. Two reviewers independently screened the contents and the agreements were achieved. Information relevant to safety was abstracted, including manifestations, severity, duration, consequences, combined treatments and causal-relation judgments by the reporter.

All data collected was considered as AEs. In monitoring data, the reporters followed the causality assessment tool of the National center for ADR monitoring which was based on the WHO-UMC causality assessment criteria [32,33]. The judgments made by the reporters were categorized into “certain”, “probable/likely”, “possible”, “unlikely”, “conditional/unclassified”, and “unassessable/unclassifiable” (File C in S1 Appendix). The first category was considered as certain ADRs, the next two categories were considered in our study as likely ADRs. The same rule was applied to the literature data and social media data as well.

We converted the AEs and ADRs into standard WHO-ART terms [34–46]. The ADR terms according to the following principles: 1) AE/ADR names in different resources that were consistent with WHO-ART terms were used directly; 2) for records in which the AE/ADR names were inconsistent with WHO-ART, the names were coded according to the detailed description of AE/ADRs; 3) for records where names and descriptions of AE/ADRs did not match, descriptions were used as main evidence of coding; 4) for records with unclear names and descriptions where coding was impossible, were labeled as “unable to code”.

The frequencies and relative frequencies of AEs, ADRs and their affected systems-organs classes were counted. Percentages were used to express the frequencies of each AE or ADR name and affected system-organ class. When more than one AEs or ADRs occurred in the same patient, each individual symptom or abnormality was counted. When one AE or ADR is classified into two or more system-organ classes according to WHO-ART, it was counted multiple times accordingly. Then we rank the top five AEs, ADRs and the affected system-organ classes. We planned to conduct disproportion analysis in monitoring system when data is sufficient. The radar chart was used to manifest the features of the distributions and proportions.

## Results

**Fig 1:**
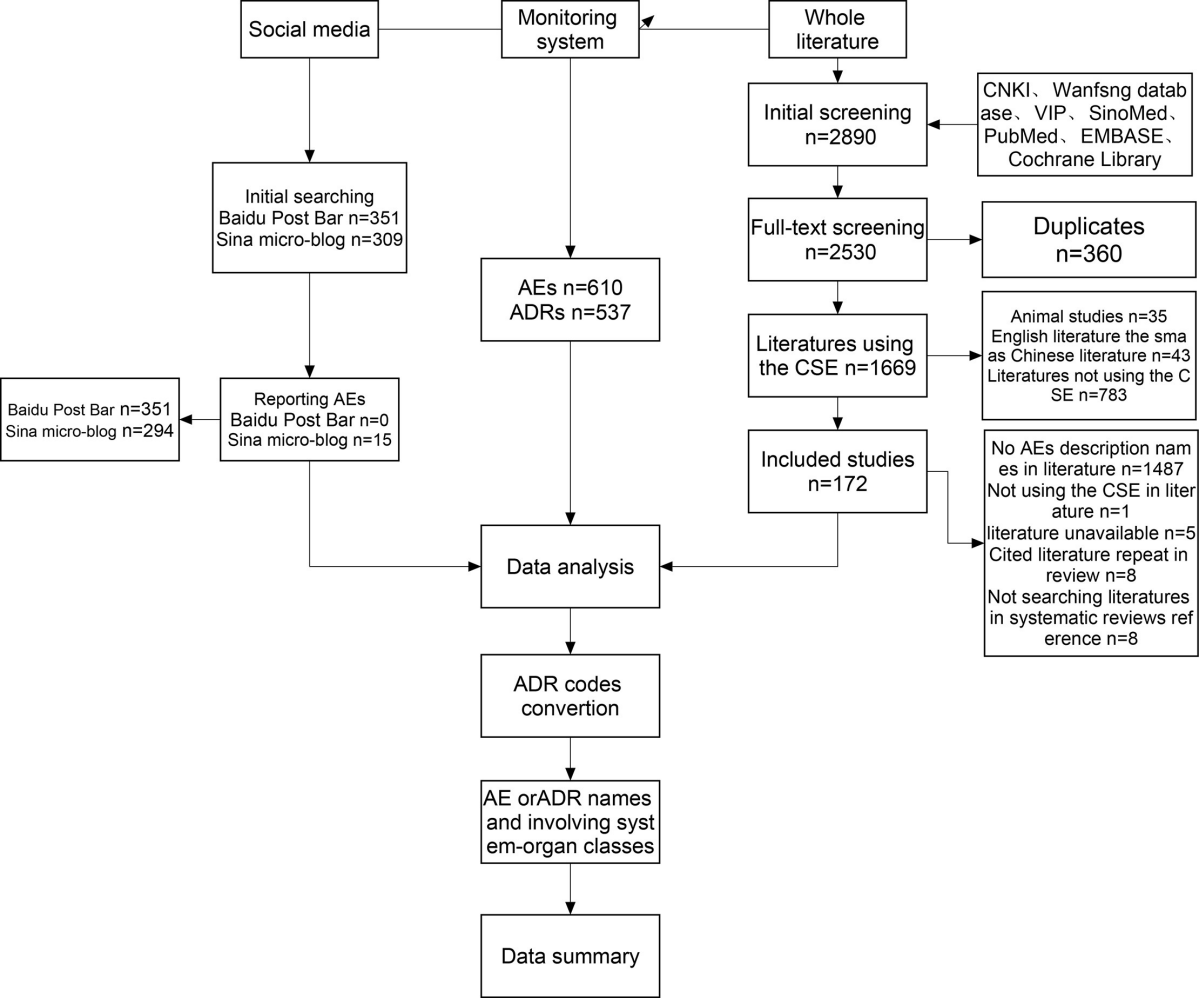
Flow diagram of the result and processes of different sources data.

Fig 1 presents the flow chart of searching and analyzing the AEs and ADRs associated with CSE based on the monitoring data, literature and social media data. And specific information extracted from them can be seen in appendix (File D in S1 Appendix).

In monitoring system, there were 610 AEs reports associated with CSE, in which 537 (88.03%) were suspected ADRs (10.49% certain). 172 Chinese language papers (63% RCTs, 27% other types of studies) were included. Studies other than RCTs were case series/reports and ADR monitoring reports and reviews. No cohort or case-control study was identified. In the literature, 5568 AEs were identified, of which 86 (1.54%) were classified as ADRs (1.54% certain). 271 AEs were identified from 108 RCTs (n = 4682). ADR rate in RCTs was 0.021%. Baidu post bar (351 themes) and Sina micro-blogs (309 posts) published a total of 660 posts. We found no useful safety information from Baidu post bar, while we identified from Sina micro-blogs 15 AEs (unassessible/unclassifiable) in which none could be judged as certain or likely ADRs due to vague descriptions. The proportion of top system-organ classes related to AEs and ADRs can be seen in Table 1.

**Table 1:** Distributions of affected system-organ classes from CFDA monitoring data and the literature.

Involved system–organ disorders	AE %(Rank)					ADR* %(Rank)			
	MD	WL	RCTs	OS	SM	MD	WL	RCTs	OS
Gastro-intestinal	40.6(1)	28.3(1)	28.0(1)	28.5(1)	16.7(1)	40.7(1)	25.5(1)	66.7(1)	22.7(1)
Autonomic nervous	17.5(2)	14.2(2)	15.3(3)	13.7(2)	11.1(3)	17.1(2)	8.5(5)	0	9.1(5)
Skin and appendages	10.0(4)	5.5(5)	3.0(8)	6.9(4)	2.78(11)	10.5(3)	12.8(2)	0	13.6(2)
Body as a whole-general	10.6(3)	7.1(4)	8.9(5)	6.2(5)	8.33(5)	10.5(3)	10.6(4)	0	11.4(4)
Central & peripheral nervous	7.3(5)	4.5(7)	4.7(7)	4.3(8)	11.11(3)	7.4(5)	12.8(2)	0	13.6(2)
Respiratory	0.8(12)	10.5(3)	16.5(2)	7.3(3)	5.56(6)	1.0(11)	8.5(5)	0	9.1(5)
Resistance mechanism	0.4(14)	4.8(6)	11.0(4)	1.4(15)	0	0.4(14)	0	0	0
Liver and biliary	3.9(6)	4.3(8)	2.5(9)	5.3(6)	5.56(6)	4.1(6)	6.4(7)	33.3(2)	4.6(7)
total number	1158	674	236	438	36	924	47	3	44

MD: Monitoring data WL: whole literature RCTs: randomized controlled trials OS: other types of studies SM: social media

**Fig 2:**
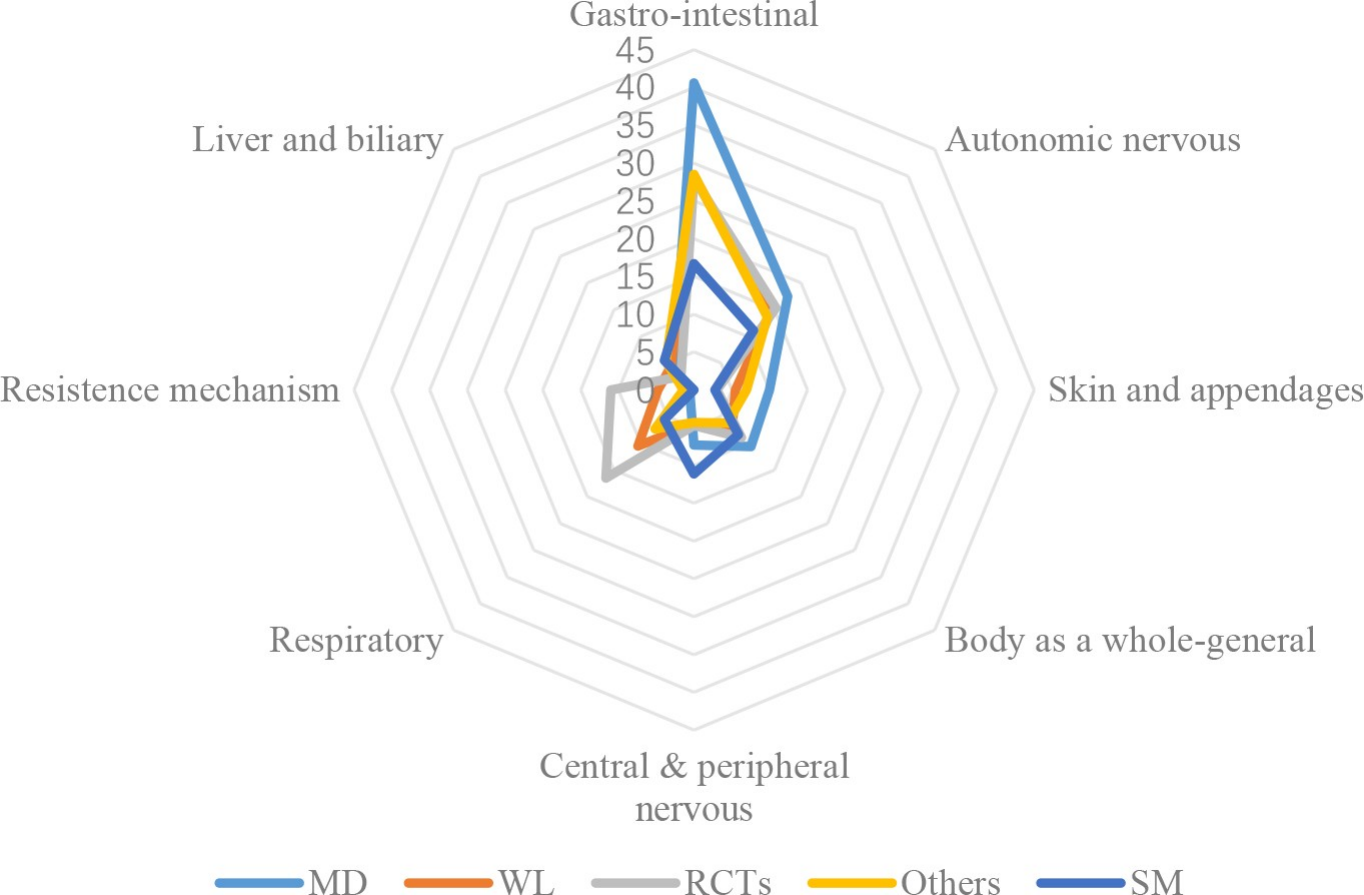
Distributions of AEs in affected system-organ classes.

Fig 2 illustrates the distributions of AEs in affected system-organ classes. Although the proportion of AEs in gastro-intestinal class was the highest in all four different data, distributions of system-organ classes reported in CFDA monitoring system were different by vision inspection from that identified in the literature. Distribution of affected system-organ classes of AEs observed in RCTs deviated most from those in CFDA monitoring data. Proportions of AEs in resistance mechanism disorder class and respiratory disorder class in the literature were much higher than those in monitoring data, while higher (10% more) proportion of AEs in the gastro-intestinal disorder class was found in monitoring data. Proportions of AEs in autonomic nervous disorders was largely similar between four different data. The central & peripheral nervous system disorder reported by social media is higher than other data.

**Fig 3:**
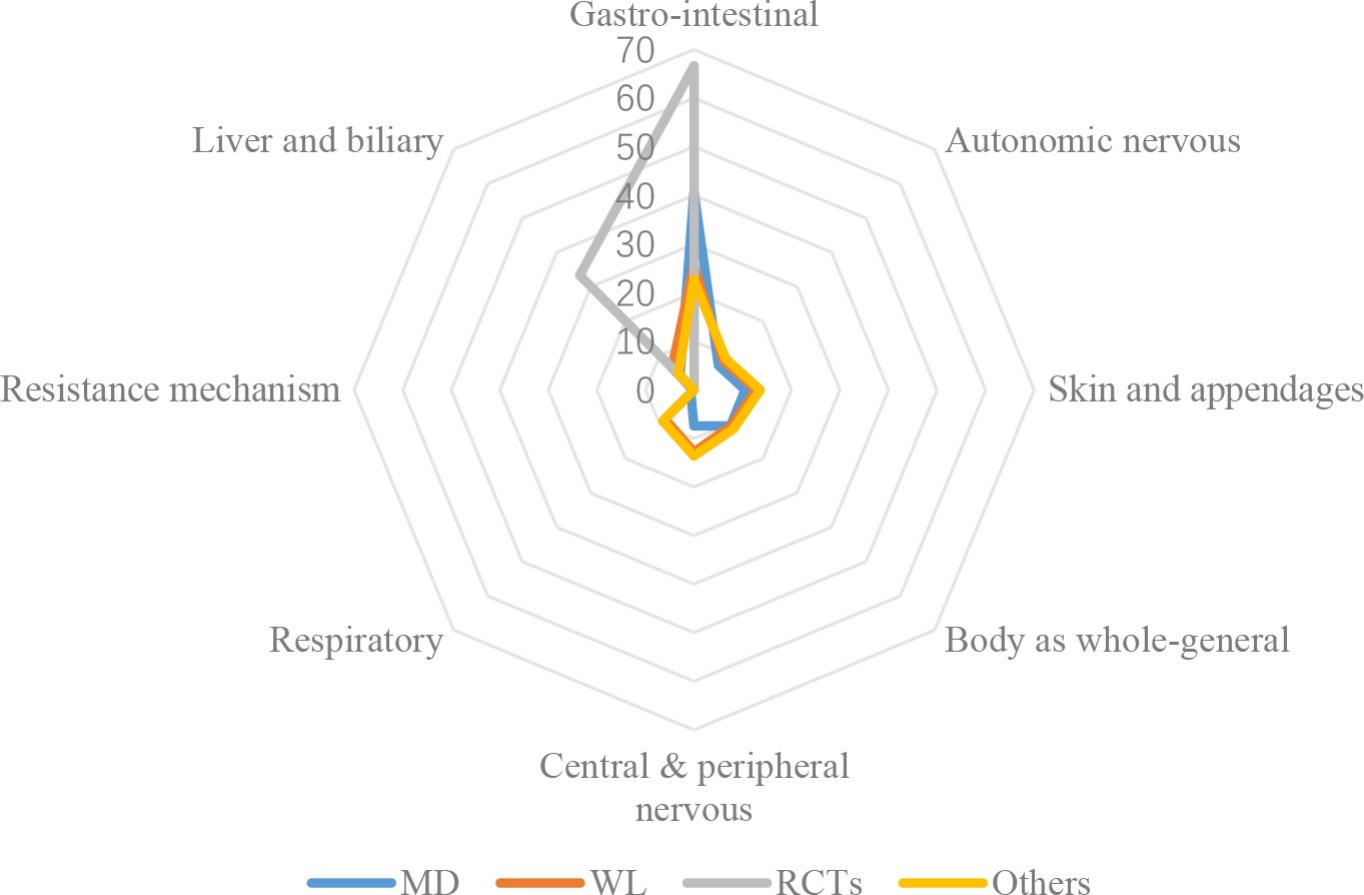
Distributions of ADRs in affected system-organs.

Proportions of AEs in gastro-intestinal class were also the highest in all different data sources; however, distribution of system-organ classes (Fig 3) in ADRs reported in CFDA monitoring system was different from that identified in the literature data. Distributions of affected system-organs of ADRs observed from RCTs, containing only gastro-intestinal class and liver and biliary class, were considerably different from CFDA monitoring data and other types of studies, which affected 7 and 6 classes respectively. Proportions of ADRs in the respiratory disorder class in the literature were much higher (9 times) than those in monitoring data. Proportions of ADRs in resistance mechanism disorder class and autonomic nervous disorder class were much higher in monitoring data than those in other types of study reports.

**Fig 4:**
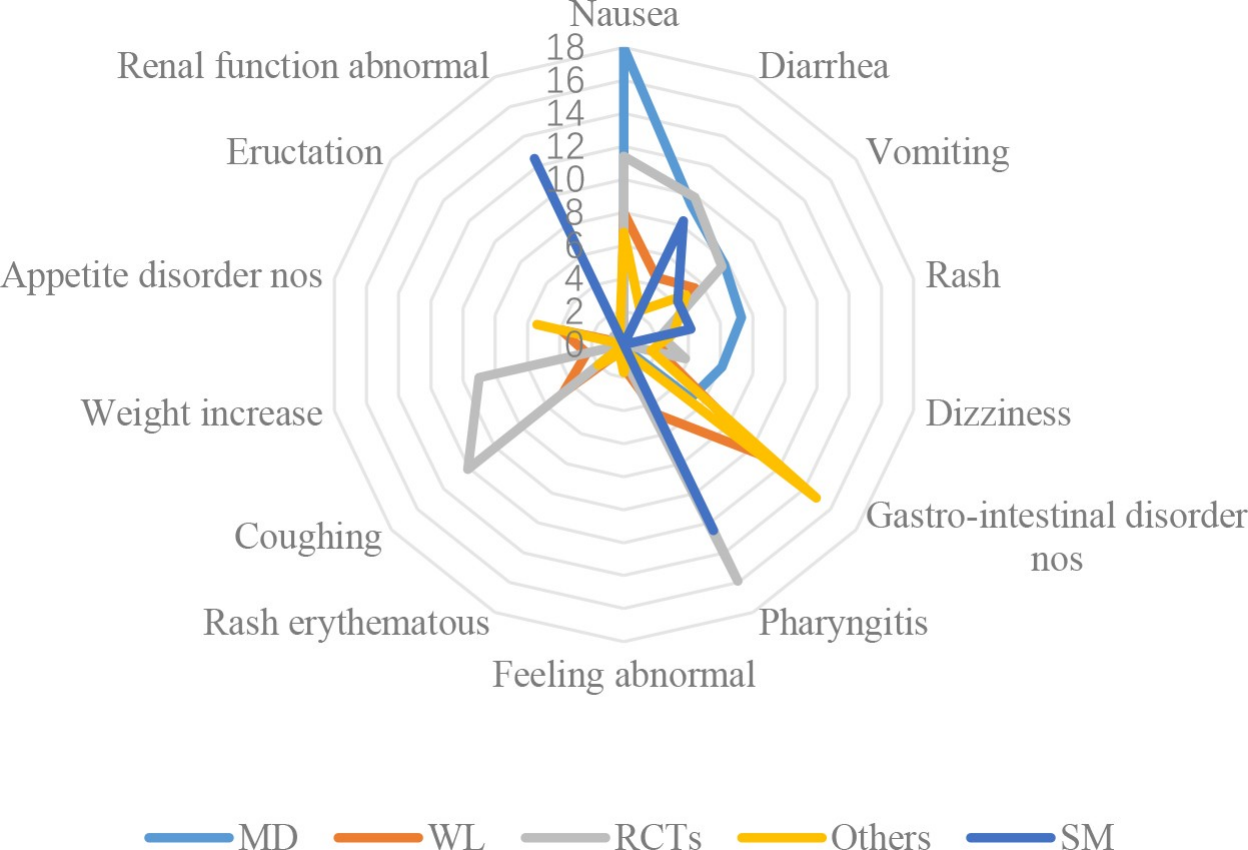
Distributions of AE names in five data sources.

In Table 2 and Fig 4, gastro-intestinal disorders including nausea, diarrhea, vomiting and non-specific disorders, were the most prevalent AEs associated with the CSE. Distributions of AEs in CFDA monitoring data were different from that identified in the literature and social media. AEs in RCT were most different from CFDA monitoring and social media data. Nausea, gastro-intestinal disorder nos (non-specific) and pharyngitis were the top names in CFDA monitoring data, whole literature/other types of studies and RCTs respectively.

**Table 2:** Distributions of AEs and ADRs in CFDA monitoring system and literatures.

AE/ADR names	AE %(Rank)					ADR %(Rank)			
	MD	WL	RCTs	OS	SM	MD	WL	RCTs	OS
Nausea	18.0(1)	8.0(2)	11.4(3)	6.8(2)	0	18.8(1)	10.8(1)	0	11.8(1)
Diarrhea	9.4(2)	4.5(6)	9.9(4)	2.3(7)	8.3(3)	8.2(2)	0	0	0
Vomiting	7.8(3)	5.5(3)	7.6(6)	4.8(4)	4.17(4)	7.9(4)	0	0	0
Rash	7.3(4)	2.9(8)	2.3(13)	3.1(5)	4.17(4)	8.1(3)	8.1(2)	0	8.8(2)
Dizziness	6.1(5)	2.3(9)	3.8(8)	1.7(10)	0	5.9(5)	5.4(4)	0	5.8(4)
Gastro-intestinal disorder nos	5.2(6)	10.8(1)	0	14.9(1)	0	5.7(6)	5.4(4)	66.7(1)	0
Pharyngitis	0.3(35)	4.7(4)	15.9(1)	0.6(23)	0	0	0	0	0
Feeling abnormal	1.0(15)	1.4(17)	0.8(16)	1.7(10)	12.5(1)	1.2(13)	8.1(2)	0	8.8(2)
Rash erythematous	0.1(55)	0.4(30)	0	0.6(23)	0	0.2(50)	5.4(4)	0	5.9(4)
Coughing	0.4(30)	4.7(4)	12.1(2)	2.0(8)	0	0.5(24)	2.7(7)	0	2.9(6)
Weight increase	0.1(55)	2.3(9)	9.0(5)	0	0	0.2(50)	0	0	0
Appetite disorder nos	0.8(16)	3.9(7)	0	5.4(3)	0	0.9(16)	2.7(7)	0	2.9(6)
Eructation	0.3(35)	0.2(44)	0.8(16)	0	0	0.2(50)	2.7(7)	33.3 (2)	0
Renal function abnormal	0	0.4 (29)	0	0.6(23)	12.5(1)	0	0	0	0
Total number	805	487	132	355	24	645	37	3	34

MD: Monitoring data WL: whole literature RCTs: randomized controlled trials OS: other types of studies SM: social media nos: non-specific

**Fig 5:**
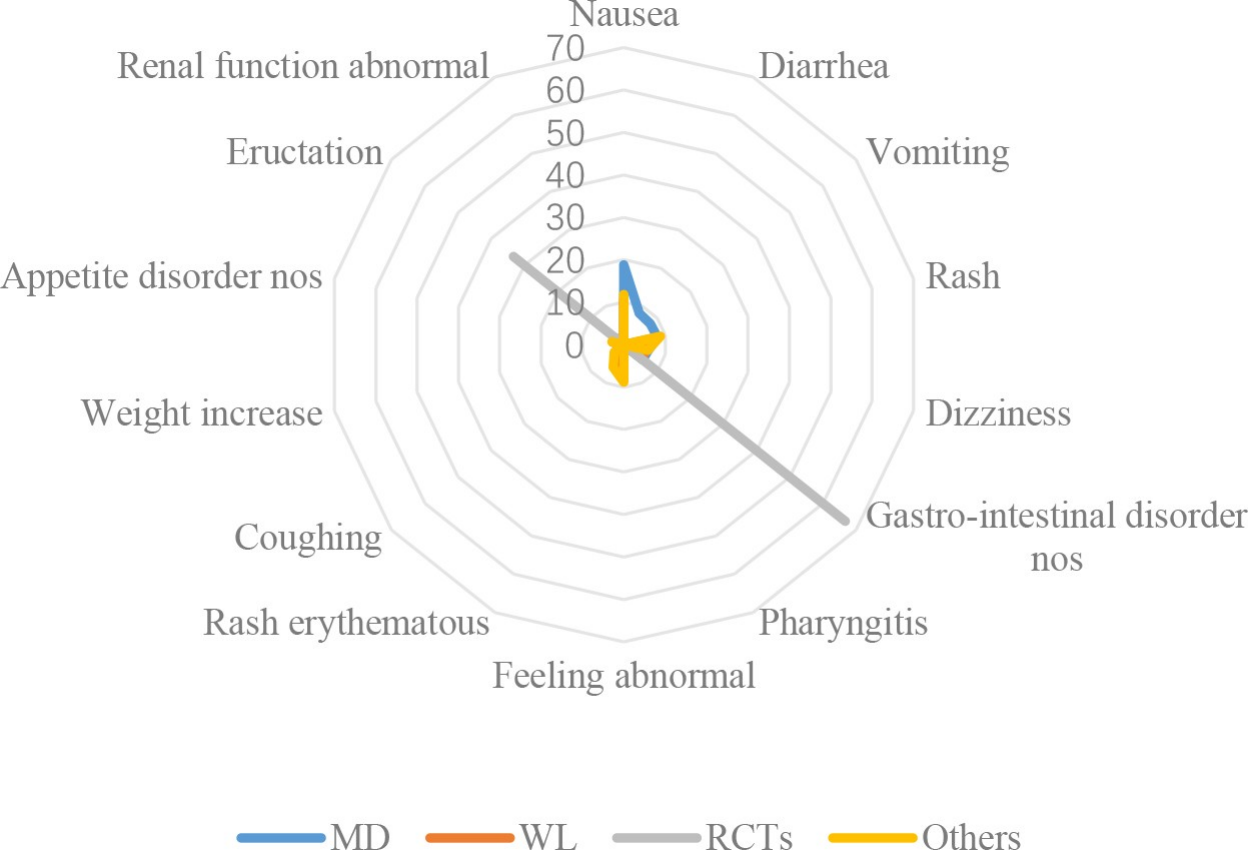
Distributions of ADR names in five data sources.

Although nausea (Fig 5) was the top ADR in CFDA monitoring data and other types of studies (mainly case series and case reports), it was not reported in the RCTs. Diarrhea and vomiting were top 2 and 3 in CFDA monitoring data, however, they were not reported in the literature. Rash and dizziness both highly prevalent (similar proportions) in CFDA monitoring data and other types of studies. Proportions of ‘feeling abnormal’ (not specified) and ‘rash erythematous’ were much higher in literatures (except RCTs) than those in CFDA monitoring data. Only gastro-intestinal disorder nos and eructation was reported in RCTs as ADRs.

Due to the inherent drawbacks of obtained data in monitoring system, we can only recognized the data-target AEs/ADRs using Chinese patent medicine CSE (a) and other AEs/ADRs using Chinese patent medicine CSE (b), but could not obtain the target AEs/ADRs in all other drugs (c) and other AEs/ADRs in all other drugs (d). So it could not be calculated the disproportionality statistics such as PRR or ROR.

## Discussion

### Summary of findings

In our example, 610 AEs reported in CFDA monitoring data were associated with CSE, of which 537 (88.03%) were ADRs (10.49% certain). 5568 AEs were identified from 172 papers (63% RCTs, 37% other types of studies including case series, case reports, ADR monitoring reports and reviews), in which 86 were ADRs (1.54% certain). 15 AEs (0 certain ADR) were identified from social media. AEs, ADRs, and their affected system-organ classes looked largely similar, mainly gastro-intestinal system disorders including nausea, diarrhea and vomiting, but different in every aspect when looking at details. Data from RCTs demonstrated most disparity.

### Strength and limitations

To our knowledge, this is the first study comparing safety data in real-world Chinese monitoring system, the literature and social media in China. We obtained national safety monitoring database (range 15 years) from professionals of the CFDA, and comprehensively searched and obtained relevant published and unpublished literatures via seven major databases, and also data from social media. The data process was complied to the PRISMA Harms checklist. The WHO-ART was adopted to standardize the names of AEs and ADRs. The Chinese drug ADR monitoring system covered all provinces with branches in cities and counties, and the number of reported ADRs has been increasing year by year.

However, there are some limitations. Our data was only for one Chinese patent medicine (CSE), thus the generalizability of our results is limited. The awareness of active reporting and monitoring is relatively low in China [47]. Safety events of CSE in the ADR monitoring database might be underreported [48]. Post-market large-sample, multicenter, well-designed clinical studies for safety of CSE were not identified. and they are especially needed to traditional Chinese medicine products [49,50]. Attempts to judge causal inference from AE to ADR were insufficient in RCTs. Natural language and unstandardized self-reports in social media were lacking important information for further judgment.

Incidence of AE or ADR could not be calculated in all data source; neither PRR nor POR can be calculated in monitoring system data. For monitoring data, the total number of people who had taken CSE in the observation period was unknown. Amongst the literature data, 14% were case series/reports. Thus, we were unable to identify the total number of patients. It is the same with data from social media. As another limitation, we did not conduct quality assessment to the original literatures, thus the quality of the included studies remained unclear. In addition, there may be very rare chance, if any, that there would be duplicates in the three data source.

### Compared to other previous works

Methodology studies on the methods of collecting information for safety evaluation found similar results with ours. TKhouri C [51], Fadini GP [52] and Wang DY [53] investigated drug safety by combined use of RCT data and pharmacovigilance data. Although their purpose of study was not to compare results from different data sources, their relevant results showed discrepancies between these two data sources. Smith K [54] compared the consistency of ADRs between Twitters, the US FDA Adverse Event Reporting System (FAERS), and the drug information databases (DIDs). They found moderate agreements between them. However, they did not compare with data from literatures. This maybe because of they only included pooled data from meta-analysis, which was not sufficient to do the comparison due to different metric of outcome results. Sonal S [55] used separately the data of observational studies and case reports in monitoring system to investigate the relationship of statins and pancreatitis and found that the results were roughly consistent.

Several methodology papers reported the limitation of using RCT data only for evaluating drug safety. RCTs are not the best suitable study design to evaluate safety [56]. Additionally, researchers may not pay enough attention to safety than effectiveness due to effectiveness being a high probability event but safety a small probability event [57]. Other limitations of RCT for evaluating safety are due to small sample size, short observation time, and strict participation selecting [58]. High quality observational studies, such as large-sample, long-term, prospective cohort study with safety assessment as its main purpose, can be considered as the most advanced evidence for the evaluation of safety evidence. In addition, when there is supporting evidence from other sources, the strength of the evidence will increase [57]. The limitations and suggestions from these methodology papers were consistent with our study results.

### Implications for future research and safety reporting

Safety conclusions based on RCTs or systematic reviews of RCTs might be more biased. And conclusions on safety in a systematic review including only RCTs need to be explained with special caution. When evaluating AEs and ADRs of interventions, for example, conducting systematic reviews for interventions or developing clinical practice guidelines, the ideal solution is to combine the real-world monitoring data, the literature data and the social media data.

We recommended that the safety data collected in the national monitoring system need to be open to the public in some way, for example, available upon reasonable request and adequate identification concealment of personal information. This will greatly support safety studies by providing real-world data source. Connecting the ADR monitoring system with HIS (Hospital Information System) data is needed to ease the data sharing to the community, decrease duplicated data an improve monitoring quality. Detailed information, such as the onset and progress of the AEs and ADRs and reasons supporting the causal inference, needs to be strengthened.

All types of literatures contribute to the evidence base for the safety of an intervention. Safety data of RCTs deviate from others most dramatically. This may due to the higher homogeneity of patients, shorter duration of treatment and follow-ups so that some specific AE/ADR cannot be found [59]. Additionally, safety information is encouraged to reported and the Consolidated Standards of Reporting Trials for reporting harms in RCTs (CONSORT Harms) [60] needs to be followed. Other types of studies also should pay attention to safety.

For systematic reviews incorporating harms of intervention, whether harms are primary outcomes or secondary outcomes, the preferred reporting items for systematic reviews and meta-analysis harms checklist: improving harms reporting in systematic reviews (PRISMA Harms) [26] should be adhered to. Guidelines with recommendations relevant to the safety of interventions need to be cautious if the evidence base contains only systematic reviews of RCTs.

It is suggested that the attention to safety information in the social media can be paid and reported in the future. And we hope to establish and develop new authoritative and reliable social media tools to collect safety information then output the safety data can be collected and dealt with better.

## Conclusion

In our study, the most prevalent AEs and ADRs, mainly gastro-intestinal system disorders including nausea, diarrhea and vomiting, in monitoring system were largely similar with those in literature and social media. But data from different sources were different if looked at details. Multiple data sources (the monitoring system, literature and social media) should be integrated to collect safety information of interventions. The distributions of AEs and ADRs from RCTs were least similar with the data from other sources. Our empirical proof is consistent with other similar studies.

## Supporting information

S1 Appendix Supporting information files. (DOCX)

## References

[1] World Health Organization. Definitions; 2019. Available from: http://www.who.int/medicines/areas/quality_safety/safety_efficacy/trainingcourses/definitions.pdf.

[2] World Health Organization. International drug monitoring: the role of national centres. TechRep Ser WHO 1972; no 498. 4625548

[3] Uppsala Monitoring Centre. The story of UMC and the WHO Programme; 2019 Available from: https://www.who-umc.org/global-pharmacovigilance/who-programme/the-story-of-umc-and-the-who-programme/

[4] Wang D, Cheng G. Annual Trend Analysis of ADR Monitoring Data. Chinese Journal of Pharmacovigilance. 2013; (05):238–241.

[5] Li W, Liu C, Li XL, Wang KP. A discussion on voluntary reporting of adverse drug reaction monitoring system. Chinese Journal of Pharmacovigilance. 2010; (01):50–52+55.

[6] Pontes H, Clément M, & Rollason V. Safety Signal Detection: The Relevance of Literature Review. Drug Safety. 2014:37(7), 471–479. 10.1007/s40264-014-0180-9 24895178

[7] Ioannidis JP, Evans SJ, Gøtzsche PC, O’Neill RT, Altman DG, Schulz K, et al Better reporting of harms in randomized trials: an extension of the CONSORT statement. Annals of internal medicine. 2004;141:781–8. 10.7326/0003-4819-141-10-200411160-00009 15545678

[8] DREAMS Trial Collaborators and West Midlands Research Collaborative. Dexamethasone versus standard treatment for postoperative nausea and vomiting in gastrointestinal surgery: randomised controlled trial (DREAMS Trial). BMJ 2017;357: j1455 10.1136/bmj.j1455 28420629 PMC5482348

[9] Liu Z, Yan S, Wu J, He L, Li N, Dong G, et al Acupuncture for Chronic Severe Functional Constipation: A Randomized Trial. Annals of internal medicine. 2016;165(11):761–769. 10.7326/M15-3118 27618593

[10] Zhao L, Chen J, Li Y, Sun X, Chang X, Zheng H, et al The Long-term Effect of Acupuncture for Migraine Prophylaxis: A Randomized Clinical Trial. JAMA internal medicine. 2017;177(4):508–515. 10.1001/jamainternmed.2016.9378 28241154

[11] Mitchell L, John P, Alfred TH, Gordon G. Harms, User's guides to the medical literature: a manual for evidence-based clinical practice. 3rd edition. JAMA evidence: McGraw-Hill Education: Columbus, US, 2015; Chapter 14(Observational Studies), 382.

[12] Onakpoya Igho J, Heneghan Carl J, Aronson Jeffrey K,Post-marketing withdrawal of 462 medicinal products because of adverse drug reactions: a systematic review of the world literature. BMC Med, 2016; 14: 10 10.1186/s12916-016-0553-2 26843061 PMC4740994

[13] Ren M. The lack of media ethics in the new media era and countermeasures Beijing institute of Graphic Communication, 2015.

[14] Han J. Baidu Post Bar and Internet Free Expression. Youth Journalist, 2008; (32): 80.

[15] Bai SY, Xiao Bl. The emotional mobilization of netizens in Sina micro-blog. Journal of Lanzhou University (Social Sciences), 2011; 39(05):60–68.

[16] Zheng WK. Present status, problems and outlook of safety evaluation for the proprietary Chinese medicine. Journal of Tianjin University of Traditional Chinese Medicine 2017; 36 (5):333–336.

[17] Xing YF, Wang BH, Huang YH. Application progress and data quality control of real-world research in clinical evaluation of traditional Chinese medicine. Global Chinese Medicine, 2018; 11 (04): 625–630.

[18] Li YY, Lei L, Xie YM. Study on centralized monitoring of 31724 cases of Dengzhan Xixin injection safety hospital. China Journal of Chinese Materia Medica,2015; 40(24):4757–4761. 27245018

[19] "Taiji Group Huoxiang Zhengqi Liquid" started a million real world studies; 2019. Available from: http://www.huaxia.com/tslj/tbgz/tbgzwz/04/5718473.html

[20] Jinshuibao capsule product introduction; 2019. Available from: http://www.jmkxjsb.com/Product-Introduction/

[21] China Health Insurance; 2019. Available from: https://www.zgylbx.com/index.php?m=content&c=index&a=lists&catid=105&steps=&search=1&pc_hash=&title=%E9%87%91%E6%B0%B4%E5%AE%9D%E8%83%B6%E5%9B%8A&k1=

[22] Chinese Pharmacopoeia Commission. Chinese pharmacopoeia: Part 1. Beijing: 2015; ISBN: 9787506773379.

[23] Chinese Medical Association Diabetes Branch Microvascular Complications Group. Diabetic Nephropathy Prevention Expert Consensus (2014 Edition). Chinese Journal of Diabete 2014; (11):792–801. 10.3760/cma.j.issn.1674-5809.2014.11.004

[24] Ministry of Health rational drug use experts committee. rational drug use guide of endocrine and metabolic diseases Beijing: People’s Medical Publishing House 2014; 103.

[25] Li G, Li ZK, Guo YB, Cheng YH, Li WS, Liu LY, Liu LY, et al; Summary of Competitiveness Report on Science and Technology of Big Brand Traditional Chinese Medicine (2018 Edition). Modern Chinese Medicine,2019; 21(01):1–19.

[26] Zorzela L, Loke YK, Ioannidis JP, Golder S, Santaguida P, Altman DG, et al; PRISMA Harms Group. PRISMA harms checklist: improving harms reporting in systematic reviews. BMJ 2016; 352: i157 10.1136/bmj.i157 26830668

[27] Hu RX, Liang N, Chai QY, Yang GY, Han XY, Xia RY, et al; PRISMA Harms checklist: improving harms reporting in systematic reviews (Part one). Chinese Journal of Pharmacovigilance, 2018; 15(06):377–384.

[28] Hu RX, Liang N, Chai QY, Yang GY, Han XY, Xia RY, et al; PRISMA Harms checklist: improving harms reporting in systematic reviews (Part two). Chinese Journal of Pharmacovigilance,2018; 15(07):445–448.

[29] Hu RX, Liang N, Chai QY, Yang GY, Han XY, Xia RY, et al; PRISMA Harms checklist: improving harms reporting in systematic reviews (Part three). Chinese Journal of Pharmacovigilance,2018, 15(08):506–512.

[30] Baidu post bar; 2019. Available from: https://tieba.baidu.com/f?kw=%E9%87%91%E6%B0%B4%E5%AE%9D&ie=utf-8&traceid=

[31] Sino micro-blog search; 2019. Available from: https://s.weibo.com/weibo?q=%E9%87%91%E6%B0%B4%E5%AE%9D&Refer=pic_weibo

[32] World Health Organization (WHO), Uppsala Monitoring Centre The use of the WHO-UMC system for standardised case causality assessment. WHO; 2019 Available from: https://www.who.int/medicines/areas/quality_safety/safety_efficacy/WHOcausality_assessment.pdf?ua=1.

[33] Adverse Drug Reaction Report and Monitoring Workbook; 2019. Available from: http://www.cdr-adr.org.cn/xzzx/hyzl/hyzl2013nd/201304/W020130426419851149382.pdf.

[34] China Food and Drug Administration, Department of Drug Safety Supervision. National Center for Adverse Drug Reaction Monitoring. Adverse drug reaction reporting and monitoring workbook. 2012; http://www.cdr-adr.org.cn/xzzx/hyzl/hyzl2013nd/201304/W020130426419851149382.pdf

[35] The WHO Adverse Reactions Terminology: Involved System-organ classes code search directory. Chinese Journal of Pharmacovigilance. 2007; 01:58–64.

[36] The WHO Adverse Reactions Terminology: Involved System-organ classes code search. Chinese Journal of Pharmacovigilance. 2007; 02:123–128.

[37] The WHO Adverse Reactions Terminology: Involved System-organ classes code search. Chinese Journal of Pharmacovigilance. 2007; 03:187–192.

[38] The WHO Adverse Reactions Terminology: Involved System-organ classes code search. Chinese Journal of Pharmacovigilance. 2007; 04:250–256.

[39] The WHO Adverse Reactions Terminology: Involved System-organ classes code search. Chinese Journal of Pharmacovigilance. 2007; 05:312–320.

[40] The WHO Adverse Reactions Terminology: Involved System-organ classes code search. Chinese Journal of Pharmacovigilance. 2007; 06:379–384.

[41] The WHO Adverse Reactions Terminology: Involved System-organ classes code search. Chinese Journal of Pharmacovigilance. 2008; 01:55–64.

[42] The WHO Adverse Reactions Terminology: Involved System-organ classes code search. Chinese Journal of Pharmacovigilance. 2008;02:122–128.

[43] The WHO Adverse Reactions Terminology: Involved System-organ classes code search. Chinese Journal of Pharmacovigilance. 2008;03:186–191.

[44] The WHO Adverse Reactions Terminology: Involved System-organ classes code search. Chinese Journal of Pharmacovigilance. 2008;04:255–256.

[45] The WHO Adverse Reactions Terminology: Involved System-organ classes code search. Chinese Journal of Pharmacovigilance. 2008;05:320.

[46] The WHO Adverse Reactions Terminology: Involved System-organ classes code search. Chinese Journal of Pharmacovigilance. 2008;06:385–386.

[47] He H, Zhu M. The progress of adverse drug reactions monitoring work in China. Journal of Liaoning University of Traditional Chinese Medicine. 2018;20(06):142–145.

[48] LI X, LI XX, Liu ZJ, Ma LX, Liu JP. Analysis of Safety Reporting in Clinical Trials on Herbal Medicine Published in Chinese Journals. Chinese Journal of Pharmacovigilance. 2010; 7(1):20–24.

[49] Zhong L, Current Situation, Challenge and Future Development of Post Marketing Evaluation on Proprietary Chinese Medicines. World Chinese Medicine 2017; 12(06):1218–1220+1225.

[50] Chan AW, Tetzlaff JM, Altman DG, Laupacis A, Gøtzsche PC, Krleža-Jerić K, et al SPIRIT 2013 statement: defining standard protocol items for clinical trials. Annals of internal medicine 2013 2 5;158(3):200–2077. 10.7326/0003-4819-158-3-201302050-00583 23295957 PMC5114123

[51] Khouri C, Lepelley M, Roustit M, Montastruc F, Humbert M, Cracowski J. Comparative Safety of Drugs Targeting the Nitric Oxide Pathway in Pulmonary Hypertension: A Mixed Approach Combining a Meta-Analysis of Clinical Trials and a Disproportionality Analysis From the World Health Organization Pharmacovigilance Database. Chest. 2018 7;154(1):136–147. 10.1016/j.chest.2017.12.008 . 29275134

[52] Fadini GP, Bonora BM, Mayur S, Rigato M, Avogaro A. Dipeptidyl peptidase-4 inhibitors moderate the risk of genitourinary tract infections associated with sodium-glucose co-transporter-2 inhibitors. Diabetes, Obesity and Metabolism. 2018 3; 20(3):740–744. 10.1111/dom.13130 29053207

[53] Wang DY, Salem J, Cohen J, Chandra S, Menzer C, Ye F, et al Fatal Toxic Effects Associated With Immune Checkpoint Inhibitors: A Systematic Review and Meta-analysis. JAMA Oncology. 2018 12 1; 4(12):1721–1728. 10.1001/jamaoncol.2018.3923 30242316 PMC6440712

[54] Smith K, Golder S, Sarker A, Loke Y, O'Connor K, Gonzalez-Hernandez G. Methods to Compare Adverse Events in Twitter to FAERS, Drug Information Databases, and Systematic Reviews: Proof of Concept with Adalimumab. Drug safety, 2018 12; 41(12):1397–1410. 10.1007/s40264-018-0707-6 30167992 PMC6223697

[55] Singh S, Loke YK. Statins and pancreatitis: a systematic review of observational studies and spontaneous case reports. Drug safety. 2006; 29(12):1123–1132. 10.2165/00002018-200629120-00004 17147459

[56] Cuervo LG, Clarke M. Balancing benefits and harms in health care. BMJ 2003;327(7406):65–66. 10.1136/bmj.327.7406.65 12855496 PMC1126436

[57] Liao X, Xie Y, Wang Y, Nicola R. To explore evidence evaluation for harm: establishing the body of evidence for harm for post-marketing traditional Chinese medicine. Chin J Chin Mater Med 2015;40(24):4723–4727. 27245012

[58] Liao X, Xie Y. Evaluating the Body of Evidence on Post-marketing Clinical Safety of Chinese Herbs. Chinese Journal of Integrated Traditional and Western Medicine. 2017; 37(01):109–114. 30695435

[59] Li M, Wang Z, Zhang XL. Discussion on the Situation of Drug Post-marketed Reassessment in China. Chin J Pharmacoepidemiol,2011;8(04):225–228.

[60] Ioannidis JP, Evans SJ, Gøtzsche PC, O'Neill RT, Altman DG, Schulz K et al; CONSORT Group. Better reporting of harms in randomized trials: an extension of the CONSORT statement. Annals of internal medicine. 2004; 141(10): 781–788. 10.7326/0003-4819-141-10-200411160-00009 15545678

