# Supplementary material for: Comparison of drug safety data obtained from the monitoring system, literature, and social media: An empirical proof from a Chinese patent medicine
Source: PLoS One. 2019 Nov 6;14(11):e0222077. doi: 10.1371/journal.pone.0222077 (PMC6834258; doi:10.1371/journal.pone.0222077)
Supplement: S1 Appendix — (DOCX) [file pone.0222077.s001.docx]

**S1 Appendix**

**Table of Contents**

[File A: 57 items included in ADR monitoring system 2](#_Toc8429992)

[File B: Search strategy. 2](#_Toc8429993)

[File C: the causality inference categories of reporter from different sources 3](#_Toc8429994)

[File D: Specific information extracted from monitoring data, literature and social media 5](#_Toc8429995)

[Table A: AEs involved system-organ classes in ADR monitoring system base 5](#_Toc8429996)

[Table B: AE names in ADR monitoring system base 7](#_Toc8429997)

[Table C: ADRs involved system-organ classes in ADR monitoring system base 9](#_Toc8429998)

[Table D: ADR names in ADR monitoring system base 10](#_Toc8429999)

[Table E: AEs names involved system-organ classes in whole literature base 12](#_Toc8430000)

[Table F: AE names in whole literature base 13](#_Toc8430001)

[Table G: ADRs involved system-organ classes in whole literature base 15](#_Toc8430002)

[Table H: ADR names in whole literature base 15](#_Toc8430003)

[Table I: AEs involved system-organ classes in RCTs base 16](#_Toc8430004)

[Table J: AE names in RCTs base 16](#_Toc8430005)

[Table K: ADRs involved system-organ classes in RCTs base 17](#_Toc8430006)

[Table L: ADR names in RCTs base 17](#_Toc8430007)

[Table M: AEs involved system-organ classes in other types of studies base 17](#_Toc8430008)

[Table N: AE names in other types of studies base 18](#_Toc8430009)

[Table O: ADRs involved system-organ classes in other types of studies base 20](#_Toc8430010)

[Table P: ADR names in other types of studies base 21](#_Toc8430011)

[Table Q: AE names in social media base 22](#_Toc8430012)

[Table R: AE involved system-organ classes in social media base 22](#_Toc8430013)

File A: 57 items included in ADR monitoring system

**1) Anonymous patient demographic information (8):**

Report ID, reporting organization type, gender, birthday, age, age unit, nationality, weight.

**2) Patient historical ADR information (3):**

History of personal ADR, history of family ADR, other important information.

**3) Patient health condition (1):**

Underlying illness.

**4) Medications received (16):**

Suspect/concomitant product, drug serial number, license number, trade name, generic name, dosage form, manufacturer name, production lot number, dosage, dosage unit, medication days, medication frequency, route of administration, medication start time, termination time, medication reasons.

**5) Description of AEs (14):**

First/following report, type of seriousness, whether is the new ADR, whether is the severe ADR,

ADR names, time of event, description of event, ADR result, sequel AEs, death time, direct cause of death, dechallenge, rechallenge, impact on the original disease.

**6) Causal inference judgments of AEs (6):**

Reporter evaluation, report unit evaluation, county evaluation, city evaluation, provincial evaluation, national evaluation.

**7) Reporter information (9):**

Report date, information source of report unit, remarks, time of national center to receive, the name of report area, time of county evaluation, time of city evaluation, time of provincial evaluation, time of national evaluation.

File B: Search strategy.

1. **Chinese database Search strategy:**

The Chinese strategy is searching the name of one certain Patent Chinese medicine in full text and ＂adverse drug reaction＂,＂side effects＂,＂toxic response＂,＂safety＂,＂adverse event＂,＂complications＂,＂reliability＂,＂toxicity＂,＂side response＂ in free word, searching exclusion ＂animal research＂ in the title, keywords or abstract.

1. **English database Search strategy:**

#1——Searching“cordyceps ssinensis” or “caterpillar fungus”in MeSH Terms.

#2——Searching “Cs-4” or “cordyceps ssinensis” or “dongchongxiacao” or “dong chong xia cao” or “C.sinensis” or “Ophiocordycep ssinensis” or “O.sinensis”in all field.

#3——Searching “cordyceps ssinensis” or “caterpillar fungus” or “dongchongxiacao” or “dong chong xiacao” or “chongcao” or “chongcao” or “C.sinesis” or “Ophiocordycep ssinensis” or “O.sinensis” or “jinshuibao” or “jin shui bao” or “Paecilomyceshepialichen”in Title/Abstract.

#4——Searching “jinshuibao” or “jin shui bao”in All field.

#5——Searching “animal NOT human”in MeSH Terms.

#6——#4 AND (#1 OR #2 OR #3) NOT #5

1. **Social media searching strategy:**

We searched “Chinese patent medicine-CSE” as the keyword in two websites. The two websites are <https://tieba.baidu.com/f?kw=%E9%87%91%E6%B0%B4%E5%AE%9D&ie=utf-8&traceid=> and <https://s.weibo.com/weibo?q=%E9%87%91%E6%B0%B4%E5%AE%9D&Refer=pic_weibo>. Then we output all information into word document so that we can do information screening according to the text content.

File C: the causality inference categories of reporter from different sources

The content of WHO-UMC causality assessment categories [1]：


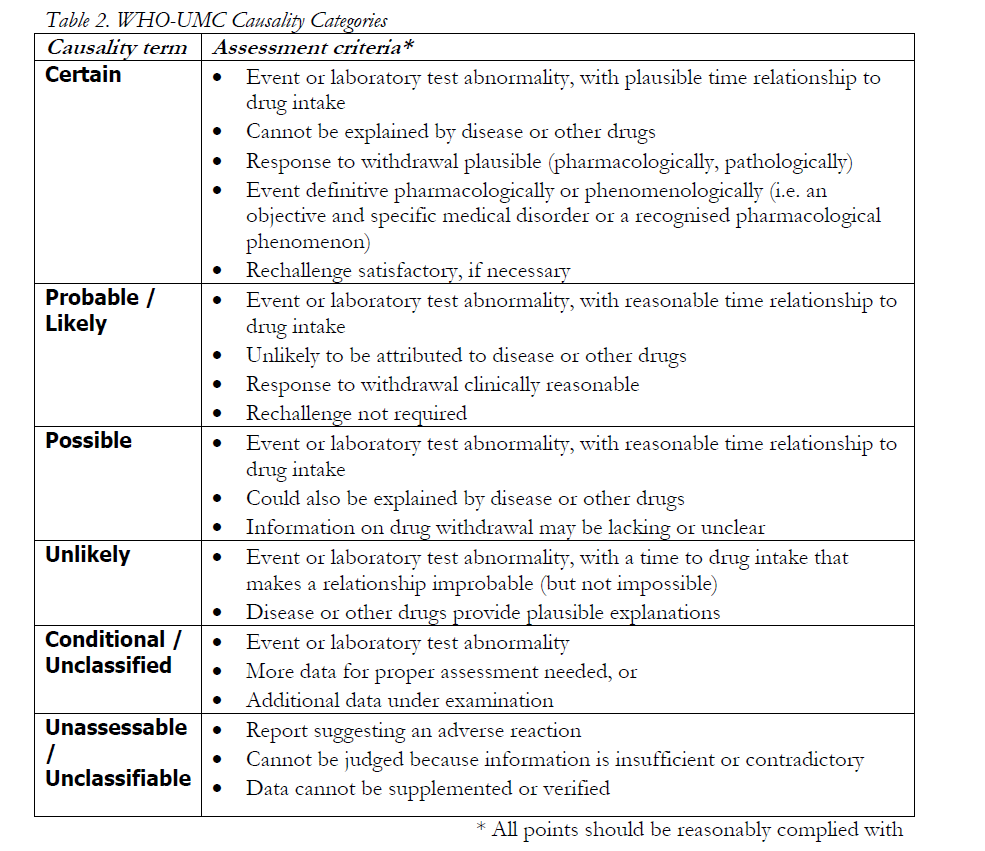


The content of ADR causality inference categories in Chinese monitoring system on page 50 [2]：

Certain: The order of administration and ADR is reasonable; the reaction stops after stopping the drug, or it is quickly relieved or improved (some ADR may occur after several days of drug withdrawal according to the immune status of the body); re-use, the ADR reappears, and may be significantly aggravated (ie, the rechallenge is positive); at the same time, there is literature evidence; and other confounding factors such as the original disease have been excluded.

Probable/Likely: There is no history of repeated medications, others is the same as "certain", or although there is a combination using of drugs, but basically the possibility of combined using of the drug can be excluded.

Possible: The medication is closely related to the time of ADR, and there is literature support; however, there is more than one drugs that can cause ADR, or the disease progression of the original disease cannot be excluded.

Unlikely: ADR is not closely related to the time of administration, and the response is not consistent with the known ADR of the drug. The development of the original disease may also have similar clinical manifestations.

Conditional/Unclassified: the content of the report is not complete, waiting for the supplement then to evaluate, or the causal relationship is difficult to determine, lack of the literature evidence.

Unassessable/Unclassifiable: There are too many missing items in the report, the causal relationship is difficult to determine, and the information cannot be added.

Reference:

[1] world Health Organization. <https://www.who.int/medicines/areas/quality_safety/safety_efficacy/WHOcausality_assessment.pdf?ua=1>.

[2]<http://www.cdr-adr.org.cn/xzzx/hyzl/hyzl2013nd/201304/W020130426419851149382.pdf>

File D: Specific information extracted from monitoring data, literature and social media

## Table A: AEs involved system-organ classes in ADR monitoring system base

| **In****volved** **system-organ classes** | **System-organ code** | **Accumulated times** | **Times proportion (%)*** | **Occurred numbers** | **proportion (%)#** |
| --- | --- | --- | --- | --- | --- |
| Skin and appendages disorders | 0100 | 116 | 10.02 | 100 | 16.39 |
| Musculo-skeletal system disorders | 0200 | 8 | 0.69 | 8 | 1.31 |
| Collagen disorders | 0300 | 0 | 0.00 | 0 | 0.00 |
| Central & peripheral nervous system disorders | 0410 | 84 | 7.25 | 82 | 13.44 |
| Autonomic nervous system disorders | 0420 | 203 | 17.53 | 199 | 32.62 |
| Vision disorders | 0431 | 1 | 0.09 | 1 | 0.16 |
| Hearing and vestibular disorders | 0432 | 0 | 0.00 | 0 | 0.00 |
| Specila senses other, disorders | 0433 | 0 | 0.00 | 0 | 0.00 |
| Psychiatric disorders | 0500 | 18 | 1.55 | 17 | 2.79 |
| Gastro-intestinal system disorders | 0600 | 470 | 40.59 | 462 | 75.74 |
| Liver and biliary system disorders | 0700 | 45 | 3.89 | 45 | 7.38 |
| Metabolic and nutritional disorders | 0800 | 16 | 1.38 | 15 | 2.46 |
| Endocrine disorders | 0900 | 0 | 0.00 | 0 | 0.00 |
| Cardiovascular disorders, general | 1010 | 17 | 1.47 | 16 | 2.62 |
| MYO, EMDO-,pericardial & valve disorders | 1020 | 2 | 0.17 | 2 | 0.33 |
| Heart rate and rhythm disorders | 1030 | 19 | 1.64 | 19 | 3.11 |
| Vascular(extracardiac) disorders | 1040 | 2 | 0.17 | 2 | 0.33 |
| Respiratory system disorders | 1100 | 9 | 0.78 | 9 | 1.48 |
| Red blood cell disorders | 1210 | 0 | 0.00 | 0 | 0.00 |
| White cell and RES disorders | 1220 | 0 | 0.00 | 0 | 0.00 |
| Platelet, bleeding & clotting disorders | 1230 | 4 | 0.35 | 4 | 0.66 |
| Urinary system disorders | 1300 | 10 | 0.86 | 9 | 1.48 |
| Reproductive disorders, male | 1410 | 2 | 0.17 | 2 | 0.3 |
| Reproductive disorders, female | 1420 | 2 | 0.17 | 2 | 0.33 |
| Foetal disorders | 1500 | 0 | 0.00 | 0 | 0.00 |
| Neonatal and infancy disorders | 1600 | 0 | 0.00 | 0 | 0.00 |
| Neoplasms | 1700 | 0 | 0.00 | 0 | 0.00 |
| Body ASA whole-general disorders | 1810 | 123 | 10.62 | 118 | 19.34 |
| Application sited disorders | 1820 | 2 | 0.17 | 2 | 0.33 |
| Resistance mechanism disorders | 1830 | 5 | 0.43 | 5 | 0.82 |
| Secondary terms | 2000 | 0 | 0.00 | 0 | 0.00 |
| Total | - | 1158 | 100.00 | 1119 | - |

Note:

* Times proportion=Accumulated times in one system-organ classes/Accumulated total times (1158)×100%

# Numbers proportion=Accumulated numbers in one system-organ classes/Accumulated total numbers(610)×100%

## Table B: AE names in ADR monitoring system base

| **Name** | **ADR code** | **Frequency** | **Proportion (%)** |
| --- | --- | --- | --- |
| Nausea | 0308 | 145 | 18.01 |
| Diarrhoea | 0205 | 76 | 9.44 |
| Vomiting | 0228 | 63 | 7.83 |
| Rash | 0027 | 59 | 7.33 |
| Dizziness | 0101 | 49 | 6.09 |
| Gastro-intestinal disorder nos | 1262 | 42 | 5.22 |
| Flatulence | 0285 | 41 | 5.09 |
| Pruritus | 0024 | 37 | 4.60 |
| Abdominal pain | 0268 | 34 | 4.22 |
| Headache | 0109 | 18 | 2.24 |
| Mouth dry | 0218 | 16 | 1.99 |
| Palpitation | 0221 | 15 | 1.86 |
| Asthenia | 0716 | 11 | 1.37 |
| Constipation | 0204 | 10 | 1.24 |
| Paraesthesia | 0137 | 8 | 0.99 |
| Rash maculo-papular | 0030 | 6 | 0.75 |
| Sweating increased | 0043 | 6 | 0.75 |
| Insomnia | 0183 | 6 | 0.75 |
| Back pain | 0717 | 6 | 0.75 |
| Periodontal disorders | 1902 | 6 | 0.75 |
| Appetite disorder nos | 1955 | 6 | 0.75 |
| Dyspepsia | 0279 | 5 | 0.62 |
| Oedema generalised | 0400 | 5 | 0.62 |
| Malaise | 0728 | 5 | 0.62 |
| Pain | 0730 | 5 | 0.62 |
| Oedema | 0398 | 4 | 0.50 |
| Face oedema | 0602 | 4 | 0.50 |
| Anaphylactoid reaction | 0714 | 4 | 0.50 |
| Fever | 0725 | 4 | 0.50 |
| Somnolence | 0197 | 3 | 0.37 |
| Coughing | 0513 | 3 | 0.37 |
| Epistaxis | 0515 | 3 | 0.37 |
| Fatigue | 0724 | 3 | 0.37 |
| Gastric function disorder | 1997 | 3 | 0.37 |
| Urticaria | 0044 | 2 | 0.25 |
| Anaesthesia locaL | 0062 | 2 | 0.25 |
| Arthralgla | 0063 | 2 | 0.25 |
| Anorexia | 0165 | 2 | 0.25 |
| Flushing | 0207 | 2 | 0.25 |
| Hypotension | 0212 | 2 | 0.25 |
| Tachycardia | 0224 | 2 | 0.25 |
| Eructation | 0283 | 2 | 0.25 |
| Gastritis | 0291 | 2 | 0.25 |
| Oesophagitis | 0309 | 2 | 0.25 |
| Stomatitis ulcerative | 0328 | 2 | 0.25 |
| SGPT increased | 0360 | 2 | 0.25 |
| Arrhythmia | 0433 | 2 | 0.25 |
| Pharyngitis | 0523 | 2 | 0.25 |
| Micturition frequency | 0606 | 2 | 0.25 |
| Breath holding | 0752 | 2 | 0.25 |
| Oedema periorbital | 1009 | 2 | 0.25 |
| Oedema genital | 1092 | 2 | 0.25 |
| Tooth ache | 1376 | 2 | 0.25 |
| Night sweats | 1898 | 2 | 0.25 |
| Acne | 0001 | 1 | 0.12 |
| Angioedema | 0003 | 1 | 0.12 |
| Dermatitis | 0007 | 1 | 0.12 |
| Stevens johnson syndrome | 0014 | 1 | 0.12 |
| Rash erythematous | 0028 | 1 | 0.12 |
| Skin discolouration | 0036 | 1 | 0.12 |
| Hyperaesthesia | 0113 | 1 | 0.12 |
| Quadriplegia | 0143 | 1 | 0.12 |
| Tremor | 0154 | 1 | 0.12 |
| Vertigo | 0158 | 1 | 0.12 |
| Emotional lability | 0177 | 1 | 0.12 |
| Vision abnormal | 0257 | 1 | 0.12 |
| Eructation | 0282 | 1 | 0.12 |
| Hiccup | 0300 | 1 | 0.12 |
| Hepatocellular damage | 0353 | 1 | 0.12 |
| Hyperuricaemia | 0385 | 1 | 0.12 |
| Obesity | 0397 | 1 | 0.12 |
| Oedema peripheral | 0401 | 1 | 0.12 |
| Thirst | 0405 | 1 | 0.12 |
| Weight decrease | 0407 | 1 | 0.12 |
| Weight increase | 0408 | 1 | 0.12 |
| Dyspnoea | 0514 | 1 | 0.12 |
| Haematuria | 0604 | 1 | 0.12 |
| Micturition disorder | 0605 | 1 | 0.12 |
| Renal pain | 0621 | 1 | 0.12 |
| Urine abnormal | 0629 | 1 | 0.12 |
| Rigors | 0731 | 1 | 0.12 |
| Hypertension aggravated | 0762 | 1 | 0.12 |
| Herpes zoster | 0862 | 1 | 0.12 |
| Halitosis | 0990 | 1 | 0.12 |
| Gingivitis | 1083 | 1 | 0.12 |
| Gastroesophageal reflux | 1149 | 1 | 0.12 |
| Dreaming abnormal | 1243 | 1 | 0.12 |
| Cannot be classified | - | 21 | 2.61 |
| Total | - | 805 | 100.00 |

## Table C: ADRs involved system-organ classes in ADR monitoring system base

| **Involved system-organ classes** | **System-organ code** | **Accumulated times** | **Times proportion (%)*** | **Occurred numbers** | **proportion (%)#** |
| --- | --- | --- | --- | --- | --- |
| Skin and appendages disorders | 0100 | 97 | 10.50 | 84 | 15.64 |
| Musculo-skeletal system disorders | 0200 | 7 | 0.76 | 7 | 1.30 |
| Collagen disorders | 0300 | 0 | 0.00 | 0 | 0.00 |
| Central & peripheral nervous system disorders | 0410 | 68 | 7.36 | 65 | 12.10 |
| Autonomic nervous system disorders | 0420 | 158 | 17.10 | 151 | 28.12 |
| Vision disorders | 0431 | 1 | 0.11 | 1 | 0.19 |
| Hearing and vestibular disorders | 0432 | 0 | 0.00 | 0 | 0.00 |
| Specila senses other, disorders | 0433 | 0 | 0.00 | 0 | 0.00 |
| Psychiatric disorders | 0500 | 16 | 1.73 | 16 | 2.98 |
| Gastro-intestinal system disorders | 0600 | 376 | 40.69 | 309 | 57.54 |
| Liver and biliary system disorders | 0700 | 38 | 4.11 | 38 | 7.08 |
| Metabolic and nutritional disorders | 0800 | 13 | 1.41 | 12 | .23 |
| Endocrine disorders | 0900 | 0 | 0.00 | 0 | 0.00 |
| Cardiovascular disorders, general | 1010 | 13 | 1.41 | 10 | 1.86 |
| MYO, EMDO-, pericardial & valve disorders | 1020 | 2 | 0.22 | 2 | 0.37 |
| Heart rate and rhythm disorders | 1030 | 16 | 1.73 | 16 | 2.98 |
| Vascular(extracardiac) disorders | 1040 | 2 | 0.22 | 2 | 0.37 |
| Respiratory system disorders | 1100 | 9 | 0.97 | 6 | 1.12 |
| Red blood cell disorders | 1210 | 0 | 0.00 | 0 | 0.00 |
| White cell and RES disorders | 1220 | 0 | 0.00 | 0 | 0.00 |
| Platelet, bleeding & clotting disorders | 1230 | 4 | 0.43 | 4 | 0.74 |
| Urinary system disorders | 1300 | 8 | 0.87 | 7 | 1.30 |
| Reproductive disorders, male | 1410 | 2 | 0.22 | 4 | 0.37 |
| Reproductive disorders, female | 1420 | 2 | 0.22 | 4 | 0.37 |
| Foetal disorders | 1500 | 0 | 0.00 | 0 | 0.00 |
| Neonatal and infancy disorders | 1600 | 0 | 0.00 | 0 | 0.00 |
| Neoplasms | 1700 | 0 | 0.00 | 0 | 0.00 |
| Body ASA whole-general disorders | 1810 | 89 | 9.63 | 80 | 14.90 |
| Application sited disorders | 1820 | 2 | 0.22 | 2 | 0.37 |
| Resistance mechanism disorders | 1830 | 4 | 0.43 | 4 | 0.74 |
| Secondary terms | 2000 | 0 | 0.00 | 0 | 0.00 |
| Total | - | 924 | 100.00 | 820 | - |

Note:

* Times proportion=Accumulated times in one system-organ classes/Accumulated total times (924)×100%

# Numbers proportion=Accumulated numbers in one system-organ classes/Accumulated total numbers(537)×100%

## Table D: ADR names in ADR monitoring system base

| **Name** | **ADR code** | **Frequency** | **Proportion (%)** |
| --- | --- | --- | --- |
| Nausea | 0308 | 121 | 18.76 |
| Diarrhoea | 0205 | 53 | 8.22 |
| Rash | 0027 | 52 | 8.06 |
| Vomiting | 0228 | 51 | 7.91 |
| Dizziness | 0101 | 38 | 5.89 |
| Gastro-intestinal disorder nos | 1262 | 37 | 5.74 |
| Flatulence | 0285 | 35 | 5.43 |
| Pruritus | 0024 | 28 | 4.34 |
| Abdominal pain | 0268 | 24 | 3.72 |
| Mouth dry | 0218 | 15 | 2.33 |
| Headache | 0109 | 14 | 2.17 |
| Palpitation | 0221 | 12 | 1.86 |
| Paraesthesia | 0137 | 8 | 1.24 |
| Constipation | 0204 | 7 | 1.09 |
| Asthenia | 0716 | 7 | 1.09 |
| Appetite disorder nos | 1955 | 6 | 0.93 |
| Rash maculo-papular | 0030 | 5 | 0.78 |
| Dyspepsia | 0279 | 5 | 0.78 |
| Back pain | 0717 | 5 | 0.78 |
| Sweating increased | 0043 | 4 | 0.62 |
| Insomnia | 0183 | 4 | 0.62 |
| Oedema generalised | 0400 | 4 | 0.62 |
| Fever | 0725 | 4 | 0.62 |
| Coughing | 0513 | 3 | 0.47 |
| Epistaxis | 0515 | 3 | 0.47 |
| Anaphylactoid reaction | 0714 | 3 | 0.47 |
| Malaise | 0728 | 3 | 0.47 |
| Pain | 0730 | 3 | 0.47 |
| Periodontal disorders | 1902 | 3 | 0.47 |
| Urticaria | 0044 | 2 | 0.31 |
| Anaesthesia local | 0062 | 2 | 0.31 |
| Arthralgla | 0063 | 2 | 0.31 |
| Anorexia | 0165 | 2 | 0.31 |
| Somnolence | 0197 | 2 | 0.31 |
| Flushing | 0207 | 2 | 0.31 |
| Tachycardia | 0224 | 2 | 0.31 |
| Gastritis | 0291 | 2 | 0.31 |
| SGPT increased | 0360 | 2 | 0.31 |
| Oedema | 0398 | 2 | 0.31 |
| Arrhythmia | 0433 | 2 | 0.31 |
| Face oedema | 0602 | 2 | 0.31 |
| Micturition frequency | 0606 | 2 | 0.31 |
| FATIGUE | 0724 | 2 | 0.31 |
| Breath holding | 0752 | 2 | 0.31 |
| Oedema periorbital | 1009 | 2 | 0.31 |
| Oedema genital | 1092 | 2 | 0.31 |
| Tooth ache | 1376 | 2 | 0.31 |
| Night sweats | 1898 | 2 | 0.31 |
| Gastric function disorder | 1997 | 2 | 0.31 |
| Acne | 0001 | 1 | 0.16 |
| Angioedema | 0003 | 1 | 0.16 |
| Dermatitis | 0007 | 1 | 0.16 |
| Erythema multiforme | 0014 | 1 | 0.16 |
| Rash erythematous | 0028 | 1 | 0.16 |
| Skin discolouration | 0036 | 1 | 0.16 |
| Hyperaesthesia | 0113 | 1 | 0.16 |
| Quadriplegia | 0143 | 1 | 0.16 |
| Tremor | 0154 | 1 | 0.16 |
| Vertigo | 0158 | 1 | 0.16 |
| Emotional lability | 0177 | 1 | 0.16 |
| Hypotension | 0212 | 1 | 0.16 |
| Vision abnormal | 0257 | 1 | 0.16 |
| Enteritis | 0282 | 1 | 0.16 |
| Eructation | 0283 | 1 | 0.16 |
| Hiccup | 0300 | 1 | 0.16 |
| Oesophagitis | 0309 | 1 | 0.16 |
| Stomatitis ulcerative | 0328 | 1 | 0.16 |
| Hepatocellular damage | 0353 | 1 | 0.16 |
| Hyperuricaemia | 0385 | 1 | 0.16 |
| Obesity | 0397 | 1 | 0.16 |
| Oedema peripheral | 0401 | 1 | 0.16 |
| Thirst | 0405 | 1 | 0.16 |
| Weight decrease | 0407 | 1 | 0.16 |
| Weight increase | 0408 | 1 | 0.16 |
| Haematuria | 0604 | 1 | 0.16 |
| Micturition disorder | 0605 | 1 | 0.16 |
| Renal pain | 0621 | 1 | 0.16 |
| Urine abnormal | 0629 | 1 | 0.16 |
| Rigors | 0731 | 1 | 0.16 |
| Hypertension aggravated | 0762 | 1 | 0.16 |
| Herpes zoster | 0862 | 1 | 0.16 |
| Dreaming abnormal | 1243 | 1 | 0.16 |
| Cannot be classified | - | 15 | 2.33 |
| Total | - | 645 | 100.00 |

## Table E: AEs names involved system-organ classes in whole literature base

| **Involved system-organ classes** | **System-organ code** | **Accumulated times** | **Times proportion (%)** |
| --- | --- | --- | --- |
| Skin and appendages disorders | 0100 | 37 | 5.49 |
| Musculo-skeletal system disorders | 0200 | 2 | 0.30 |
| Collagen disorders | 0300 | 0 | 0.00 |
| Central & peripheral nervous system disorders | 0410 | 30 | 4.45 |
| Autonomic nervous system disorders | 0420 | 96 | 14.24 |
| Vision disorders | 0431 | 0 | 0.00 |
| Hearing and vestibular disorders | 0432 | 0 | 0.00 |
| Specila senses other, disorders | 0433 | 0 | 0.00 |
| Psychiatric disorders | 0500 | 21 | 3.12 |
| Gastro-intestinal system disorders | 0600 | 191 | 28.34 |
| Liver and biliary system disorders | 0700 | 29 | 4.30 |
| Metabolic and nutritional disorders | 0800 | 22 | 3.26 |
| Endocrine disorders | 0900 | 18 | 2.67 |
| Cardiovascular disorders, general | 1010 | 9 | 1.34 |
| MYO, EMDO-, pericardial & valve disorders | 1020 | 7 | 1.04 |
| Heart rate and rhythm disorders | 1030 | 10 | 1.48 |
| Vascular(extracardiac) disorders | 1040 | 5 | 0.74 |
| Respiratory system disorders | 1100 | 71 | 10.53 |
| Red blood cell disorders | 1210 | 5 | 0.74 |
| White cell and RES disorders | 1220 | 13 | 1.93 |
| Platelet, bleeding & clotting disorders | 1230 | 13 | 1.93 |
| Urinary system disorders | 1300 | 11 | 1.63 |
| Reproductive disorders, male | 1410 | 0 | 0.00 |
| Reproductive disorders, female | 1420 | 1 | 0.15 |
| Foetal disorders | 1500 | 0 | 0.00 |
| Neonatal and infancy disorders | 1600 | 0 | 0.00 |
| Neoplasms | 1700 | 0 | 0.00 |
| Body ASA whole-general disorders | 1810 | 48 | 7.12 |
| Application sited disorders | 1820 | 2 | 0.30 |
| Resistance mechanism disorders | 1830 | 32 | 4.75 |
| Secondary terms | 2000 | 1 | 0.15 |
| Total | - | 674 | 100.00 |

## Table F: AE names in whole literature base

| **Name** | **ADR code** | **Frequency** | **Proportion (%)** |
| --- | --- | --- | --- |
| Gastro-intestinal disorder nos | 1262 | 53 | 10.88 |
| Nausea | 0308 | 39 | 8.01 |
| Vomiting | 0228 | 27 | 5.54 |
| Coughing | 0513 | 23 | 4.72 |
| Pharyngitis | 0523 | 23 | 4.72 |
| Diarrhoea | 0205 | 22 | 4.52 |
| Appetite disorder nos | 1955 | 19 | 3.90 |
| Rash | 0027 | 14 | 2.87 |
| Dizziness | 0101 | 11 | 2.26 |
| Weight increase | 0408 | 11 | 2.26 |
| Pruritus | 0024 | 11 | 2.26 |
| Constipation | 0204 | 10 | 2.05 |
| Flatulence | 0285 | 10 | 2.05 |
| Palpitation | 0221 | 9 | 1.85 |
| Death | 0722 | 8 | 1.64 |
| Leucopenia | 0908 | 8 | 1.64 |
| Paraesthesia | 0137 | 7 | 1.44 |
| Headache | 0109 | 6 | 1.23 |
| Asthenia | 0716 | 6 | 1.23 |
| Hepatic function abnormal | 0348 | 5 | 1.03 |
| Upper resp tract infection | 0543 | 5 | 1.03 |
| Acne | 0001 | 4 | 0.82 |
| Abdominal pain | 0268 | 4 | 0.82 |
| GI haemorrhage | 0294 | 4 | 0.82 |
| SGPT increased | 0360 | 4 | 0.82 |
| Marrow depression | 0561 | 4 | 0.82 |
| Creatinine clearance decreased | 0598 | 3 | 0.62 |
| Pneumonitis | 1141 | 3 | 0.62 |
| Rash erythematous | 0028 | 2 | 0.41 |
| Rash maculo-papular | 0030 | 2 | 0.41 |
| Injection site pain | 0057 | 2 | 0.41 |
| Quadriplegia | 0143 | 2 | 0.41 |
| Somnolence | 0197 | 2 | 0.41 |
| Oedema generalised | 0400 | 2 | 0.41 |
| Phlebitis | 0455 | 2 | 0.41 |
| Dyspnoea | 0514 | 2 | 0.41 |
| Anaphylactoid reaction | 0714 | 2 | 0.41 |
| Back pain | 0717 | 2 | 0.41 |
| Fever | 0725 | 2 | 0.41 |
| Infection | 0736 | 2 | 0.41 |
| Infection bacterial | 0738 | 2 | 0.41 |
| Renal function abnormal | 0619 | 2 | 0.41 |
| Alopecia | 0002 | 1 | 0.21 |
| Angioedema | 0003 | 1 | 0.21 |
| Convulsions grand mal | 0095 | 1 | 0.21 |
| Insomnia | 0183 | 1 | 0.21 |
| Hypertension | 0210 | 1 | 0.21 |
| Tachycardia | 0224 | 1 | 0.21 |
| Eructation | 0283 | 1 | 0.21 |
| Peritonitis | 0320 | 1 | 0.21 |
| Hepatic function abnormal | 0348 | 1 | 0.21 |
| Hyponatraemia | 0392 | 1 | 0.21 |
| Oedema | 0398 | 1 | 0.21 |
| Oedema peripheral | 0401 | 1 | 0.21 |
| Embolism limb | 0449 | 1 | 0.21 |
| Embolism pulmonary | 0451 | 1 | 0.21 |
| Purpura allergic | 0460 | 1 | 0.21 |
| Cardiac failure | 0496 | 1 | 0.21 |
| Pleural effusion | 0524 | 1 | 0.21 |
| Sputum increased | 0541 | 1 | 0.21 |
| Anaemia | 0544 | 1 | 0.21 |
| Granulocytopenia | 0572 | 1 | 0.21 |
| Face oedema | 0602 | 1 | 0.21 |
| Nocturia | 0611 | 1 | 0.21 |
| Oliguria | 0612 | 1 | 0.21 |
| Urine abnormal | 0629 | 1 | 0.21 |
| Menstrual disorder | 0657 | 1 | 0.21 |
| Malaise | 0728 | 1 | 0.21 |
| Hypochloraemia | 0816 | 1 | 0.21 |
| Hypoproteinaemia | 0827 | 1 | 0.21 |
| Herpes zoster | 0862 | 1 | 0.21 |
| Pigmentation abnormal | 0973 | 1 | 0.21 |
| Hepatorenal syndrome | 1103 | 1 | 0.21 |
| Asthma | 1367 | 1 | 0.21 |
| Malnutrition | 1972 | 1 | 0.21 |
| Cannot be classified | - | 73 | 14.99 |
| Total | - | 487 | 100.00 |

## Table G: ADRs involved system-organ classes in whole literature base

| **Involved system-organ classes** | **System-organ code** | **Accumulated times** | **Times proportion (%)** |
| --- | --- | --- | --- |
| Skin and appendages disorders | 0100 | 6 | 12.77 |
| Musculo-skeletal system disorders | 0200 | 0 | 0.00 |
| Collagen disorders | 0300 | 0 | 0.00 |
| Central & peripheral nervous system disorders | 0410 | 6 | 12.77 |
| Autonomic nervous system disorders | 0420 | 4 | 8.51 |
| Vision disorders | 0431 | 0 | 0.00 |
| Hearing and vestibular disorders | 0432 | 0 | 0.00 |
| Specila senses other, disorders | 0433 | 0 | 0.00 |
| Psychiatric disorders | 0500 | 1 | 2.13 |
| Gastro-intestinal system disorders | 0600 | 12 | 25.53 |
| Liver and biliary system disorders | 0700 | 3 | 6.38 |
| Metabolic and nutritional disorders | 0800 | 1 | 2.13 |
| Endocrine disorders | 0900 | 0 | 0.00 |
| Cardiovascular disorders, general | 1010 | 1 | 2.13 |
| MYO, EMDO-, pericardial & valve disorders | 1020 | 1 | 2.13 |
| Heart rate and rhythm disorders | 1030 | 1 | 2.13 |
| Vascular(extracardiac) disorders | 1040 | 1 | 2.13 |
| Respiratory system disorders | 1100 | 4 | 8.51 |
| Red blood cell disorders | 1210 | 0 | 0.00 |
| White cell and RES disorders | 1220 | 0 | 0.00 |
| Platelet, bleeding & clotting disorders | 1230 | 1 | 2.13 |
| Urinary system disorders | 1300 | 0 | 0.00 |
| Reproductive disorders, male | 1410 | 0 | 0.00 |
| Reproductive disorders, female | 1420 | 0 | 0.00 |
| Foetal disorders | 1500 | 0 | 0.00 |
| Neonatal and infancy disorders | 1600 | 0 | 0.00 |
| Neoplasms | 1700 | 0 | 0.00 |
| Body ASA whole-general disorders | 1810 | 5 | 10.64 |
| Application sited disorders | 1820 | 0 | 0.00 |
| Resistance mechanism disorders | 1830 | 0 | 0.00 |
| Secondary terms | 2000 | 0 | 0.00 |
| Total | - | 47 | 100.00 |

## Table H: ADR names in whole literature base

| **Name** | **ADR code** | **Frequency** | **Proportion (%)** |
| --- | --- | --- | --- |
| Pruritus | 0024 | 1 | 2.70 |
| Rash | 0027 | 3 | 8.11 |
| Rash erythematous | 0028 | 2 | 5.41 |
| Dizziness | 0101 | 2 | 5.41 |
| Paraesthesia | 0137 | 3 | 8.11 |
| Somnolence | 0197 | 1 | 2.70 |
| Palpitation | 0221 | 1 | 2.70 |
| Abdominal pain | 0268 | 1 | 2.70 |
| Eructation | 0283 | 1 | 2.70 |
| Flatulence | 0285 | 1 | 2.70 |
| Nausea | 0308 | 4 | 10.81 |
| Oedema generalised | 0400 | 1 | 2.70 |
| Purpura allergic | 0460 | 1 | 2.70 |
| Coughing | 0513 | 1 | 2.70 |
| Anaphylactoid reaction | 0714 | 1 | 2.70 |
| Asthenia | 0716 | 1 | 2.70 |
| Malaise | 0728 | 1 | 2.70 |
| Gastro- intestinal disorder nos | 1262 | 2 | 5.41 |
| Asthma | 1367 | 1 | 2.70 |
| Appetite disorder nos | 1955 | 1 | 2.70 |
| Cannot be classified | - | 7 | 18.92 |
| Total | - | 37 | 100.00 |

## Table I: AEs involved system-organ classes in RCTs base

| **Involved system-organ classes** | **System-organ code** | **Accumulated times** | **Times proportion (%)** |
| --- | --- | --- | --- |
| Gastro-intestinal system disorders | 0600 | 66 | 27.97 |
| Respiratory system disorders | 1100 | 39 | 16.53 |
| Autonomic nervous system disorders | 0420 | 36 | 15.25 |
| Resistance mechanism disorders | 1830 | 26 | 11.02 |
| Body as a whole-general disorders | 1810 | 21 | 8.90 |
| Metabolic and nutritional disorders | 0800 | 15 | 6.36 |
| Central & peripheral nervous system disorders | 0410 | 11 | 4.66 |
| Skin and appendages disorders | 0100 | 7 | 2.97 |
| Liver and biliary system disorders | 0700 | 6 | 2.54 |
| Heart rate and rhythm disorders | 1030 | 4 | 1.69 |
| Musculo-skeletal system disorders | 0200 | 1 | 0.42 |
| Cardiovascular disorders, general | 1010 | 1 | 0.42 |
| MYO-, EMDO-, pericardial & valve disorders | 1020 | 1 | 0.42 |
| White cell and RES disorders | 1220 | 1 | 0.42 |
| Urinary system disorders | 1300 | 1 | 0.42 |
| Total | - | 236 | 100 |

## Table J: AE names in RCTs base

| **Name** | **ADR code** | **Frequency** | **Proportion (%)** |
| --- | --- | --- | --- |
| Pharyngitis | 0523 | 21 | 15.91 |
| Coughing | 0513 | 16 | 12.12 |
| Nausea | 0308 | 15 | 11.36 |
| Diarrhoea | 0205 | 13 | 9.85 |
| Weight increase | 0408 | 11 | 8.33 |
| Vomiting | 0228 | 10 | 7.58 |
| Constipation | 0204 | 8 | 6.06 |
| Flatulence | 0285 | 5 | 3.79 |
| Dizziness | 0101 | 5 | 3.79 |
| Palpitation | 0221 | 4 | 3.03 |
| Headache | 0109 | 4 | 3.03 |
| Acne | 0001 | 4 | 3.03 |
| Rash | 0027 | 3 | 2.27 |
| Asthenia | 0716 | 2 | 1.52 |
| Upper resp tract infection | 0543 | 2 | 1.52 |
| Back pain | 0717 | 1 | 0.76 |
| Quadriplegia | 0143 | 1 | 0.76 |
| Death | 0722 | 1 | 0.76 |
| Oedema | 0398 | 1 | 0.76 |
| Creatinine clearance decreased | 0598 | 1 | 0.76 |
| Hypertension | 0210 | 1 | 0.76 |
| Paraesthesia | 0137 | 1 | 0.76 |
| Leucopenia | 0908 | 1 | 0.76 |
| Eructation | 0283 | 1 | 0.76 |
| Total | - | 132 | 100.00 |

## Table K: ADRs involved system-organ classes in RCTs base

| **Involved system-organ classes** | **System-organ code** | **Accumulated times** | **Times proportion (%)** |
| --- | --- | --- | --- |
| Gastro-intestinal system disorders | 0600 | 2 | 66.67 |
| Liver and biliary system disorders | 0700 | 1 | 33.33 |
| Total | - | 3 | 100 |

## Table L: ADR names in RCTs base

| Name | ADR code | Frequency | Proportion (%) |
| --- | --- | --- | --- |
| Gastro-intestinal disorder nos | 0908 | 2 | 66.67 |
| Eructation | 0283 | 1 | 33.33 |
| Total | - | 3 | 100.00 |

## Table M: AEs involved system-organ classes in other types of studies base

| **Involved system-organ classes** | **System-organ code** | **Accumulated times** | **Times proportion (%)** |
| --- | --- | --- | --- |
| Gastro-intestinal system disorders | 0600 | 125 | 28.54 |
| Autonomic nervous system disorders | 0420 | 60 | 13.70 |
| Respiratory system disorders | 1100 | 32 | 7.31 |
| Skin and appendages disorders | 0100 | 30 | 6.85 |
| Body as a whole- general disorders | 1810 | 27 | 6.16 |
| Liver and biliary system disorders | 0700 | 23 | 5.25 |
| Psychiatric disorders | 0500 | 21 | 4.79 |
| Central & peripheral nervous system disorders | 0410 | 19 | 4.34 |
| Endocrine disorders | 0900 | 18 | 4.11 |
| Platelet, bleeding & clotting disorders | 1230 | 13 | 2.97 |
| White cell and res disorders | 1220 | 12 | 2.74 |
| Urinary system disorders | 1300 | 10 | 2.28 |
| Cardiovascular disorders, general | 1010 | 8 | 1.83 |
| Metabolic and nutritional disorders | 0800 | 7 | 1.60 |
| MYO-, EMDO-, pericardial & valve disorders | 1020 | 6 | 1.37 |
| Heart rate and rhythm disorders | 1030 | 6 | 1.37 |
| Resistance mechanism disorders | 1830 | 6 | 1.37 |
| Vascular(extracardiac)disorders | 1040 | 5 | 1.14 |
| Red blood cell disorders | 1210 | 5 | 1.14 |
| Application site disorders | 1820 | 2 | 0.46 |
| Musculo-skeletal system disorders | 0200 | 1 | 0.23 |
| Reproductive disorders, female | 1420 | 1 | 0.23 |
| Secondary terms | 2000 | 1 | 0.23 |
| Collagen disorders | 0300 | 0 | 0.00 |
| Vision disorders | 0431 | 0 | 0.00 |
| Hearing and vestibular disorders | 0432 | 0 | 0.00 |
| Specila senses other, disorders | 0433 | 0 | 0.00 |
| Reproductive disorders, male | 1410 | 0 | 0.00 |
| Foetal disorders | 1500 | 0 | 0.00 |
| Neonatal and infancy disorders | 1600 | 0 | 0.00 |
| Neoplasms | 1700 | 0 | 0.00 |
| Total | - | 438 | 100.00 |

## Table N: AE names in other types of studies base

| **Name** | **ADR code** | **Frequency** | **Proportion (%)** |
| --- | --- | --- | --- |
| Gastro- intestinal disorder nos | 1262 | 53 | 14.93 |
| Nausea | 0308 | 24 | 6.76 |
| Appetite disorder nos | 1955 | 19 | 5.35 |
| Vomiting | 0228 | 17 | 4.79 |
| Rash | 0027 | 11 | 3.10 |
| Pruritus | 0024 | 10 | 2.82 |
| Diarrhoea | 0205 | 8 | 2.25 |
| Death | 0722 | 7 | 1.97 |
| Leucopenia | 0908 | 7 | 1.97 |
| Coughing | 0513 | 7 | 1.97 |
| Dizziness | 0101 | 6 | 1.69 |
| Feeling abnormal | 0137 | 6 | 1.69 |
| Flatulence | 0285 | 5 | 1.41 |
| Palpitation | 0221 | 5 | 1.41 |
| Hepatic function abnormal | 0348 | 5 | 1.41 |
| Asthenia | 0716 | 4 | 1.13 |
| Abdominal pain | 0268 | 4 | 1.13 |
| GI haemorrhage | 0294 | 4 | 1.13 |
| SGPT increased | 0360 | 4 | 1.13 |
| Marrow depression | 0561 | 4 | 1.13 |
| Upper resp tract infection | 0543 | 3 | 0.85 |
| Pneumonia | 1141 | 3 | 0.85 |
| Pharyngitis | 0523 | 2 | 0.56 |
| Constipation | 0204 | 2 | 0.56 |
| Headache | 0109 | 2 | 0.56 |
| Creatinine clearance decreased | 0598 | 2 | 0.56 |
| Rash erythematous | 0028 | 2 | 0.56 |
| Rash maculo-papular | 0030 | 2 | 0.56 |
| Injection site pain | 0057 | 2 | 0.56 |
| Somnolence | 0197 | 2 | 0.56 |
| Oedema generalised | 0400 | 2 | 0.56 |
| Phlebitis | 0455 | 2 | 0.56 |
| Dyspnoea | 0514 | 2 | 0.56 |
| Anaphylactoid reaction | 0714 | 2 | 0.56 |
| Fever | 0725 | 2 | 0.56 |
| Infection | 0736 | 2 | 0.56 |
| Infection bacterial | 0738 | 2 | 0.56 |
| Renal function abnormal | 0619 | 2 | 0.56 |
| Quadriplegia | 0143 | 1 | 0.28 |
| Back pain | 0717 | 1 | 0.28 |
| Alopecia | 0002 | 1 | 0.28 |
| Angioedema | 0003 | 1 | 0.28 |
| Pruritus | 0024 | 1 | 0.28 |
| Convulsions grand mal | 0095 | 1 | 0.28 |
| Insomnia | 0183 | 1 | 0.28 |
| Diarrhoea | 0205 | 1 | 0.28 |
| Tachycardia | 0224 | 1 | 0.28 |
| Peritonitis | 0320 | 1 | 0.28 |
| Hepatic function abnormal | 0348 | 1 | 0.28 |
| Hyponatraemia | 0392 | 1 | 0.28 |
| Oedema peripheral | 0401 | 1 | 0.28 |
| Embolism limb | 0449 | 1 | 0.28 |
| Embolism pulmonary | 0451 | 1 | 0.28 |
| Purpura allergic | 0460 | 1 | 0.28 |
| Cardiac failure | 0496 | 1 | 0.28 |
| Pleural effusion | 0524 | 1 | 0.28 |
| Sputum increased | 0541 | 1 | 0.28 |
| Anaemia | 0544 | 1 | 0.28 |
| Granulocytopenia | 0572 | 1 | 0.28 |
| Face oedema | 0602 | 1 | 0.28 |
| Nocturia | 0611 | 1 | 0.28 |
| Oliguria | 0612 | 1 | 0.28 |
| Urine abnormal | 0629 | 1 | 0.28 |
| Menstrual disorder | 0657 | 1 | 0.28 |
| Malaise | 0728 | 1 | 0.28 |
| Hypochloraemia | 0816 | 1 | 0.28 |
| Hypoproteinaemia | 0827 | 1 | 0.28 |
| Herpes zoster | 0862 | 1 | 0.28 |
| Pigmentation abnormal | 0973 | 1 | 0.28 |
| Hepatorenal syndrome | 1103 | 1 | 0.28 |
| Asthma | 1367 | 1 | 0.28 |
| Malnutrition | 1972 | 1 | 0.28 |
| Weight increase | 0408 | 0 | 0.00 |
| Acne | 0001 | 0 | 0.00 |
| Hypertension | 0210 | 0 | 0.00 |
| Eructation | 0283 | 0 | 0.00 |
| Oedema | 0398 | 0 | 0.00 |
| Cannot be classified | - | 72 | 20.56 |
| Total | - | 355 | 100.00 |

## Table O: ADRs involved system-organ classes in other types of studies base

| **Involved system-organ classes** | **System-organ code** | **Accumulated times** | **Times proportion (%)** |
| --- | --- | --- | --- |
| Gastro-intestinal system disorders | 0600 | 10 | 22.73 |
| Skin and appendages disorders | 0100 | 6 | 13.64 |
| Central & peripheral nervous system disorders | 0410 | 6 | 13.64 |
| Body as a whole-general disorders | 1810 | 5 | 11.36 |
| Autonomic nervous system disorders | 0420 | 4 | 9.09 |
| Respiratory system disorders | 1100 | 4 | 9.09 |
| Liver and biliary system disorders | 0700 | 2 | 4.55 |
| Psychiatric disorders | 0500 | 1 | 2.27 |
| Metabolic and nutritional disorders | 0800 | 1 | 2.27 |
| Cardiovascular disorders, general | 1010 | 1 | 2.27 |
| Myo-, emdo-, pericardial & valve disorders | 1020 | 1 | 2.27 |
| Heart rate and rhythm disorders | 1030 | 1 | 2.27 |
| Vascular (extracardiac) disorders | 1040 | 1 | 2.27 |
| Platelet, bleeding & clotting disorders | 1230 | 1 | 2.27 |
| Musculo-skeletal system disorders | 0200 | 0 | 0.00 |
| Collagen disorders | 0300 | 0 | 0.00 |
| Vision disorders | 0431 | 0 | 0.00 |
| Hearing and vestibular disorders | 0432 | 0 | 0.00 |
| Specila senses other, disorders | 0433 | 0 | 0.00 |
| Endocrine disorders | 0900 | 0 | 0.00 |
| Red blood cell disorders | 1210 | 0 | 0.00 |
| White cell and res disorders | 1220 | 0 | 0.00 |
| Urinary system disorders | 1300 | 0 | 0.00 |
| Reproductive disorders, male | 1410 | 0 | 0.00 |
| Reproductive disorders, female | 1420 | 0 | 0.00 |
| Foetal disorders | 1500 | 0 | 0.00 |
| Neonatal and infancy disorders | 1600 | 0 | 0.00 |
| Neoplasms | 1700 | 0 | 0.00 |
| Application site disorders | 1820 | 0 | 0.00 |
| Resistance mechanism disorders | 1830 | 0 | 0.00 |
| Secondary terms | 2000 | 0 | 0.00 |
| Total | - | 44 | 100.00 |

## Table P: ADR names in other types of studies base

| **Name** | **ADR code** | **Frequency** | **Proportion (%)** |
| --- | --- | --- | --- |
| Nausea | 0308 | 4 | 11.76 |
| Rash | 0027 | 3 | 8.82 |
| Paraesthesia | 0137 | 3 | 8.82 |
| Rash erythematous | 0028 | 2 | 5.88 |
| Dizziness | 0101 | 2 | 5.88 |
| Gastro- intestinal disorder nos | 1262 | 0 | 0.00 |
| Pruritus | 0024 | 1 | 2.94 |
| Somnolence | 0197 | 1 | 2.94 |
| Palpitation | 0221 | 1 | 2.94 |
| Abdominal pain | 0268 | 1 | 2.94 |
| Eructation | 0283 | 0 | 0.00 |
| Flatulence | 0285 | 1 | 2.94 |
| Oedema generalised | 0400 | 1 | 2.94 |
| Purpura allergic | 0460 | 1 | 2.94 |
| Coughing | 0513 | 1 | 2.94 |
| Anaphylactoid reaction | 0714 | 1 | 2.94 |
| Asthenia | 0716 | 1 | 2.94 |
| Malaise | 0728 | 1 | 2.94 |
| Asthma | 1367 | 1 | 2.94 |
| Appetite disorder nos | 1955 | 1 | 2.94 |
| Cannot be classified | - | 7 | 20.59 |
| Total | - | 34 | 100.00 |

## Table Q: AE names in social media base

| **Names** | **ADR code** | **Frequency** | **Proportion (%)** |
| --- | --- | --- | --- |
| Feeling abnormal | 0317 | 3 | 12.50 |
| Renal function abnormal | 0619 | 3 | 12.50 |
| Diarrhoea | 0205 | 2 | 8.33 |
| Hepatic function abnormal | 0348 | 2 | 8.33 |
| Haematuria | 0604 | 2 | 8.33 |
| Rash | 0027 | 1 | 4.17 |
| Headache | 0109 | 1 | 4.17 |
| Anorexia | 0165 | 1 | 4.17 |
| Insomnia | 0183 | 1 | 4.17 |
| Vomiting | 0228 | 1 | 4.17 |
| Abdominal pain | 0268 | 1 | 4.17 |
| Oedema | 0398 | 1 | 4.17 |
| Hyperthyroidism | 0415 | 1 | 4.17 |
| Dyspnoea | 0514 | 1 | 4.17 |
| Anaemia | 0544 | 1 | 4.17 |
| Colitis haemorrhagic | 1180 | 1 | 4.17 |
| Cannot be classified | - | 1 | 4.17 |
| Total | - | 24 | 100.00 |

## Table R: AE involved system-organ classes in social media base

| Involved system-organ classes | Involved system-organ class code | Frequency | Proportion (%) |
| --- | --- | --- | --- |
| Gastro-intestinal system disorders | 0600 | 6 | 16.67 |
| Urinary system disorders | 1300 | 5 | 13.89 |
| Central & peripheral nervous system disorders | 0410 | 4 | 11.11 |
| Autonomic nervous system disorders | 0420 | 4 | 11.11 |
| Body as a whole- general disorders | 1810 | 3 | 8.33 |
| Psychiatric disorders | 0500 | 2 | 5.56 |
| Liver and biliary system disorders | 0700 | 2 | 5.56 |
| Cardiovascular disorders, general | 1010 | 2 | 5.56 |
| Respiratory system disorders | 1100 | 2 | 5.56 |
| Platelet, bleeding & clotting disorders | 1230 | 2 | 5.56 |
| Skin and appendages disorders | 0100 | 1 | 2.78 |
| Metabolic and nutritional disorders | 0800 | 1 | 2.78 |
| Endocrine disorders | 0900 | 1 | 2.78 |
| Red blood cell disorders | 1210 | 1 | 2.78 |
| Total | - | 36 | 100.00 |
